# Comparison of the burden of self-reported bacterial sexually transmitted infections among men having sex with men across 68 countries on four continents

**DOI:** 10.1186/s12889-023-15946-8

**Published:** 2023-05-30

**Authors:** Ulrich Marcus, Maria Veras, Jordi Casabona, Carlos F. Caceres, Nathan Lachowsky, Susanne B. Schink, Axel J. Schmidt

**Affiliations:** 1 Department of Infectious Disease Epidemiology, Robert Koch-Institute, Berlin, Germany; 2 Faculdade de Ciências Médicas da Santa Casa de São Paulo, Rua Dr Cesario Mota Jr 61, São Paulo, SP 01221-020 Brazil; 3 Scientific Director of CEEISCAT, Barcelona, Spain; 4 Centre for Interdisciplinary Studies in Sexuality, AIDS and Society, Universidad Peruana Cayetano Heredia, Lima, Peru; 5 School of Public Health and Social Policy, University of Victoria, Victoria, BC Canada; 6 Community Based Research Centre Society, Vancouver, BC Canada; 7 Sigma Research, London School of Hygiene and Tropical Medicine, London, UK

**Keywords:** Bacterial sexually transmitted diseases, Burden of illness, Health problem, international, Male homosexuality, Community surveys

## Abstract

**Background:**

Men who have sex with men (MSM) are in general more vulnerable to sexually transmitted infections (STIs) than the heterosexual men population. However, surveillance data on STI diagnoses lack comparability across countries due to differential identification of MSM, diagnostic standards and methods, and screening guidelines for asymptomatic infections.

**Methods:**

We compared self-reported overall diagnostic rates for syphilis, gonorrhea, and chlamydia infections, and diagnostic rates for infections that were classified to be symptomatic in the previous 12 months from two online surveys. They had a shared methodology, were conducted in 68 countries across four continents between October 2017 and May 2018 and had 202,013 participants.

**Results:**

Using multivariable multilevel regression analysis, we identified age, settlement size, number of sexual partners, condom use for anal intercourse, testing frequency, sampling rectal mucosa for extragenital testing, HIV diagnosis, and pre-exposure prophylaxis use as individual-level explanatory variables. The national proportions of respondents screened and diagnosed who notified some or all of their sexual partners were used as country-level explanatory variables. Combined, these factors helped to explain differences in self-reported diagnosis rates between countries. The following differences were not explained by the above factors: self-reported syphilis diagnoses were higher in Latin America compared with Europe, Canada, Israel, Lebanon, and the Philippines (aORs 2.30 – 3.71 for symptomatic syphilis compared to Central-West Europe); self-reported gonorrhea diagnoses were lower in Eastern Europe and in Latin America compared with all other regions (aORs 0.17-0.55 and 0.34 - 0.62 for symptomatic gonorrhea compared to Central-West Europe); and self-reported chlamydia diagnoses were lower in Central East and Southeast Europe, South and Central America, and the Philippines (aORs 0.25 - 0.39 for symptomatic chlamydia for Latin American subregions compared to Central West Europe).

**Conclusions:**

Possible reasons for differences in self-reported STI diagnosis prevalence likely include different background prevalence for syphilis and syndromic management without proper diagnosis, and different diagnostic approaches for gonorrhea and chlamydia.

**Supplementary Information:**

The online version contains supplementary material available at 10.1186/s12889-023-15946-8.

## Introduction

Data on syphilis, gonorrhea, and chlamydia diagnoses in men having sex with men (MSM) across different countries and world regions are not readily available. Data from surveillance systems on sexually transmitted infections (STIs) are often incomplete, and same-sex sexual practices of patients with an STI are often not routinely collected and reported. Sexual health services are organized differently across countries, with various screening policies for STIs, and specific to MSM, and varying availability and affordability of diagnostic tools [1].

STIs have been identified as important co-factors for HIV acquisition and transmission [2–4]. Behavioral change in response to the HIV epidemic contributed to an unprecedented decline of syphilis, gonorrhea and chlamydia diagnoses among MSM in the late 1980s and early 1990s [5–7]. However, this trend reversed in the late 1990s: the number of bacterial STI diagnoses among MSM in high-income countries has steadily increased in the last two decades [8–10].

Three developments may have contributed to these increases:

Increasing partner numbers: After an initial decline in partner numbers, these started to increase during the 1990s, possibly due to treatment advances and growing confidence in manageability of HIV transmission risks [11–13]. The emergence and adoption of new technologies to seek and find sexual partners online revolutionized partner seeking, in particular among MSM living in areas with few or no gay venues [14–17].

Declining condom use: Advances in HIV treatment and increased awareness of HIV status facilitated HIV-seroadaptive prevention methods [18–23]. HIV-serosorting, i.e. the selection of partners and decisions not to use condoms with partners of the same HIV status, increased awareness and knowledge of undetectable equals untransmissible (U = U), and lastly the introduction of oral HIV chemoprophylaxis (PrEP) reduced the perceived necessity to rely purely on condoms for prevention of HIV.

Increased STI detection: Mucosal infections with *Neisseria gonorrhoeae* (NG) and *Chlamydia trachomatis* (CT) are more easily detected by highly sensitive nucleic acid amplification tests (NAAT), which has increased the number of asymptomatic, largely extragenital, infections diagnosed. Many high-income countries increasingly screen MSM for asymptomatic NG/CT infections in urine specimens and in urethral, rectal and pharyngeal swabs [24].

We sought to investigate the differences in self-reported diagnosis rates for syphilis, gonorrhea and chlamydia among MSM who participated in large pan-European and Latin American internet surveys conducted in 2017–18. While the limitation of self-reported diagnosis data is well-known [25–27], our experience with self-reported diagnosis data from both surveys shows that they are highly comparable with surveillance data and bio-behavioral survey data, and are particularly suitable for international rankings [28–30]. Our survey used the same methods and definitions while being also available in up to 33 languages. We hypothesized that the differences in overall self-reported diagnosis rates for these three STIs are largely explained by different screening approaches, also reflecting differences in access to health care including prevention services, by different sexual behaviors (e.g. numbers of partners and condom use), and possibly by the extent and effectiveness of partner notification (PN) following the diagnosis of an STI. We expected screening approaches to impact detection and diagnosis rates, especially for gonorrhea and chlamydia. Due to the sizeable proportions of asymptomatic infections, the actual infection prevalence is difficult to measure. Instead, we used overall self-reported diagnosis rates and classified symptomatic diagnoses. Symptomatic diagnoses reflect infection/disease burden better than overall diagnosis rates because they depend less on access to and uptake of asymptomatic screening. It could also be argued that measuring and comparing asymptomatic gonorrhea or chlamydia infections that usually clear within a few weeks without clinical sequelae for MSM might not represent the most critical disease burden [31–33]. From an individual point of view, a comparison of infection burden is only meaningful for symptomatic infections with gonorrhea and chlamydia. From a public health perspective, infection burden encompasses symptomatic as well as asymptomatic infections because both are transmissible, assuming screening for asymptomatic infections reduces infection prevalence. For gonorrhea and chlamydia, this has only been suggested by mathematic modeling [34, 35] and no empirical studies. However, the validity of modeling predictions is uncertain, since none of the modeling approaches included immune responses to asymptomatic infections. Models that include temporary immunity predict substantially lower impacts of therapeutic STI interventions, most likely because the direct benefit of the intervention in the short term is offset by a longer-term reduction in the prevalence of immunity [36].

The primary goal of our analysis was to establish a ranking for the (self-reported) STI disease burden among MSM in the countries in which we recruited sufficient participants. A secondary goal was to assess the impact of screening activities on the variability of self-reported STI diagnosis rates, particularly for gonorrhea and chlamydia, and to explore whether and how the association of the already known behavioral and diagnostic factors with STI diagnosis probabilities differ for the three STIs.

## Methods

### Study population

The study population recruited by the European MSM Internet Survey in 2017–18 (EMIS) and the Latin American MSM Internet Survey in 2018 (LAMIS) were men living in Europe, Israel, Lebanon, the Philippines, Canada, Mexico, Central America, or South America, who are sexually attracted to men and/or had sex with men, and who indicated that they understood the nature and purpose of the study and consented to take part. To provide consent, respondents had to confirm by checking a box on the introductory page that explained the study’s purpose and procedures. EMIS respondents had to indicate whether they were old enough to legally have sex with men in the country they lived in, while LAMIS respondents had to indicate whether they were 18 years or older. The need for parental or guardian consent for the participation of minors in EMIS countries where minors were old enough to legally have sex with men was waived by the Observational Research Ethics Committee at the London School of Hygiene and Tropical Medicine. The information provided on the introductory pages for both surveys where participants provided informed consent is available as Additional File 1.

### Recruitment and questionnaires

The detailed methods of EMIS-2017 (EMIS) and LAMIS-2018 (LAMIS) have been reported elsewhere [37, 38]. In summary, EMIS and LAMIS were multi-language, internet-based, self-completion surveys for MSM living in Europe, or Latin America running from 13 Oct 2017 to 31 Jan 2018 (EMIS), and from 24 Jan to 13 May 2018 (LAMIS). The EMIS data collection additionally included a few non-European countries, namely Israel (part of the WHO European region), Lebanon, Canada, and the Philippines. Both surveys used the same questionnaire to collect data about sexual behaviors, prevention behaviors related to HIV, self-assessed STI-testing behaviors including the recency of the last test, diagnostic procedures (blood test or genital or anal swab) and self-reported STI diagnoses, including gonorrhea, chlamydia and syphilis, and whether symptoms were present at the last STI test. EMIS was available in 33 languages across 50 countries, LAMIS in three languages across 18 countries. Participants were recruited through trans-national dating apps (PlanetRomeo, Grindr and Hornet accounted for 69% of participants in both surveys collectively, other dating platforms and apps for another 9%), through Facebook, Twitter, Instagram (8%), and through a variety of local online promotion means, mostly through website banners (8%). No financial incentives were given to participants. No personal identifying information (including IP addresses) were collected. Further background information, including all 33 language versions of the questionnaires, is available at www.emis-project.eu. Ethics approval was granted by the Ethics Committee of the London School of Hygiene and Tropical Medicine (reference 14,421/RR/8805) for EMIS, and by the committees of the Universidad Peruana Cayetano Heredia (612-19-17), the Salvador Allende School of Public Health, Faculty of Medicine, University of Chile (009-2017), Santa Casa de Misericórdia de São Paulo, Brazil (2,457,744), the National Committee for Health Ethics, Guatemala (39-2017) and the Faculty of Psychology and Neuroscience of the University of Maastricht, The Netherlands (18-01-12-2017 ) for LAMIS [39].

### Dependent variables

#### Primary outcomes

All men were asked ‘Have you ever been diagnosed with syphilis?’ Men who answered yes, were asked ‘When were you last diagnosed with syphilis?’ and offered a scale to indicate how recently this had happened. Identical questions were asked for ‘gonorrhea’ and ‘chlamydia or LGV’. We grouped syphilis diagnosed in the past 24 h, seven days, four weeks, six months and 12 months as “syphilis diagnosed in the past 12 months”. Gonorrhea and chlamydia/LGV were grouped accordingly [39].

If tested within the past 12 months, the question if symptoms were present at the time of the last STI test allowed us to classify self-reported STI diagnoses as symptomatic or as asymptomatic as long as they were diagnosed at the last test. If more than one infection was diagnosed and symptoms were reported, we classified them as symptomatic if only gonorrhea and chlamydia had been diagnosed – and as unclassifiable if syphilis as well as gonorrhea and/or chlamydia had been diagnosed, as we did not know which infection caused the symptoms. For a better understanding of the link between survey questions and outcome definitions see Additional Fig. S1.

This study thus focuses on six independent outcomes, all measured with a 12-months recall period: any self-reported diagnosis of (1) syphilis, (2) gonorrhea, and (3) chlamydia, and any symptomatic diagnosis of (4) syphilis, (5) gonorrhea, and (6) chlamydia.

### Independent variables

#### Country grouping

Our results cover 68 countries overall, including four European microstates that were counted with the surrounding or neighboring countries, and Albania, Kosovo and Montenegro were combined to one region, as the national sample sizes were each smaller than 100. Thus, the total number of ‘countries’ shown in the figures is 62.

To reduce complexity but retain geographically coherent and culturally similar entities, most of the 68 countries covered by EMIS and LAMIS were combined to form sub-regions, only Brazil, Canada, Mexico, and the Philippines were not grouped with other countries. The sub-regions are: *Central America* (Costa Rica, Guatemala, Honduras, Nicaragua, Panama, El SaIvador); *Andean countries* (Bolivia, Ecuador, Colombia, Peru; also including Venezuela and Suriname, the only Dutch-speaking country in the region); *Southern Cone* (Argentina, Chile, Uruguay, Paraguay); *Southwest Europe* (Andorra, Spain, Italy, Malta, Portugal, San Marino); *West Europe* (Belgium, France, Ireland, Netherlands, Monaco, United Kingdom), *Northwest Europe* (Denmark, Finland, Iceland, Norway, Sweden), *Northeast Europe* (Estonia, Latvia, Lithuania); *Central-East Europe* (Czech Republic, Hungary, Poland, Slovakia, Slovenia); *East Europe* (Belarus, Moldova, Russia, Ukraine); *Southeast Europe* (Albania, Bulgaria, Romania, Bosnia-Herzegovina, Croatia, Kosovo, Montenegro, North Macedonia, Serbia, Cyprus, Greece, Turkey); *Middle East* (Israel, Lebanon). The mainly German-speaking countries of *Central-West Europe* (Austria, Germany, Liechtenstein, Luxembourg, Switzerland) are the sub-region with the largest number of participants and form the reference group.

#### Survey artefacts

The wording in the French translation for STI diagnoses, while technically correct, may have been interpreted by some French-speaking respondents as having undergone a test rather than having had a positive test result. This problem affects all European countries with large sub-samples in French, notably France, Belgium, Switzerland, in descending order for decreasing proportions of French speakers, as reflected by the disproportionate increase of self-reported STI diagnoses in these countries (questionnaires submitted in French: France: 93%, Belgium: 36%, Switzerland: 19%). In Canada, a country with a French-speaking minority, the potential for misunderstanding appears to have been smaller. To control for a potential overestimation of STI diagnoses in French questionnaires, a binary language variable (French – any other language) was constructed. We further controlled for major discrepancies (discrepant answers for age, steady partners, or non-steady partners), using a binary variable. Such discrepancies occur when respondents either give random answers or always select e.g. the first response option [39].

#### Sample composition

In the multivariable regression models age was included as a categorical variable with the age groups ‘younger than 25’, ’25–29’, ’30–39’, ’40–49’, and ‘50 and older’; HIV diagnosis as a binary variable; settlement size as an ordinal variable with the five categories ‘rural, villages (< 10,000 inhabitants)’, ‘small cities or towns (10,000–99,999)’, ‘medium-sized cities or towns (100,000–499,999)’, ‘big cities or towns (500,000–999,999)’, and ‘very big cities or towns (one million and more)’; financial coping in the three categories ‘comfortable’, ‘neither struggling nor comfortable’, and ‘struggling’.

#### STI testing behavior

As a proxy for testing frequency, the recency of the last STI test was categorized as ‘within the last 4 weeks’, ‘1–6 months ago’, ‘screening 6–12 months ago’, ‘no screening’. Respondents reporting symptoms at the last STI test were included in ‘no screening’ which must be considered when interpreting the results when the ‘no screening’ group is used for reference.

#### Mucosal sites sampled

*Neisseria gonorrhea* and *Chlamydia trachomatis* are frequently detected in MSM in anal and pharyngeal swabs; however, the collection of pharyngeal swabs was not queried. Since extragenital testing increases the number of diagnoses, we included anal swabbing as a binary variable in the regression models for gonorrhea and chlamydia.

#### Sexual behavior

As the number of sexual partners is a major determinant for STI transmission, we included an ordinal variable for the number of overall sexual partners in the previous 12 months: None or one, 2–4, 5–7, 8–10, 11–20, > 20 partners. Based on a question on condom use during anal intercourse with non-steady partners in the previous 12 months, we constructed an ordinal variable for anal intercourse and condom use: No anal intercourse (AI) with non-steady partners or no non-steady partner in the previous 12 months; always using condoms with non-steady partners; respectively mostly, sometimes, seldom and not using condoms. Since the number of missing answers for condom use was relatively high and clearly not at random, we decided to create and include a category of missing answers for this variable. The use of HIV pre-exposure prophylaxis (PrEP) was categorized as current daily use, current on-demand use, former PrEP use, and no experience with PrEP. Participation in sex with multiple concurrent partners was categorized in a binary variable as having had multiple concurrent partners during the last sex with a non-steady partner, or not.

#### Country-level STI screening rates

To capture STI diagnostic procedures and extragenital testing, two country-level variables were constructed and categorized by quartiles for screening practices for syphilis and gonorrhea/chlamydia: (1) being screened for syphilis, defined as reporting no symptoms at last test and reporting a blood-based test as part of STI-testing in the previous 12 months; (2) being screened for gonorrhea/chlamydia, defined as reporting no symptoms at last test and reporting a test based on a genital specimen (urine or urethral swab) *and* an anal swab as part of STI-testing in the previous 12 months (see Additional Fig. S1). Respondents with an STI diagnosis that was unclassifiable or classified symptomatic were subtracted from numerator and denominator. These two variables were used for all three respective primary outcomes [39].

#### Country-level partner notification rates for syphilis and gonorrhea

survey respondents who reported a diagnosis with syphilis and/or gonorrhea within the previous 12 months were asked immediately after the diagnosis question, ‘The last time you were diagnosed with syphilis (or gonorrhea, respectively), did you or your healthcare provider inform your recent sexual partners that they also needed a test/treatment?’ with the response options ‘No, none of them’; ‘Yes, some of them’; ‘Yes, all of them’; and ‘I don’t remember’. The response options were binarized in ‘Yes, some or all of them’ and ‘No, none of them, or I don’t remember’, and proportions of partner notification (PN) for syphilis and gonorrhea were calculated for each country and ranked by quartiles. We included this country level variable hypothesizing that increasing levels of PN after an STI diagnosis in a country might reduce the probability of the diagnosis of this STI, because more transmission chains are interrupted.

### Statistics

For continuous variables we calculated mean and standard deviation (SD), or median and interquartile range (IQR). For nominal and ordinal variables, we calculated count and percentage.

Based on theoretical assumptions, a list of variables that are potentially associated with the dependent variables was developed for each outcome variable. We used a bivariate approach and a two-level multilevel logistic regression model with a random intercept at country level in order to identify the list of significantly associated variables with the dependent variables. The random component accounts for the hierarchical nature of the data. All available cases across both surveys were analyzed, in the multivariable model cases with missing data on any of the included variables were excluded, with condom use being the only exception (see above).

We developed models for each dependent variable: the first step in model building was to enter stepwise those individual-level independent variables that were statistically significantly associated with the dependent variable for each model (based on bivariate analysis). Age was also included as a potential confounder. Variables from the significantly associated pool were then included sequentially in the multivariable analysis. The variables were added to the null model one by one retaining those variables significant at p < 0.05. The final models were then estimated with the pool of significantly associated variables. The likelihood ratio test was used to compare the new model with the nested model to establish the model improvement. For all statistical tests, significance was indicated by p < 0.05. The final model estimated the adjusted odds ratios (aORs) and the corresponding 95% confidence interval (95%-CI) for factors associated with the dependent variable. Analyses were carried out using Stata Version 17.1 (College Station, TX: StataCorp LP) [39].

#### Assessment of the relative impact of screening activities on the variance of diagnoses

In principle, there is no method to delineate exactly how much a specific variable or set of variables explains the variance in multilevel multivariable analysis. As a surrogate, we compared Efron’s pseudo R^2^ in simple multivariable logistic regression models between the full models and models only including the three screening-related variables screening recency (1), number of sampled sites (2), and proportion of participants that have been screened in a country (3) for gonorrhea and chlamydia diagnoses, and with a model including (1) and (3) for syphilis diagnoses.

## Results

### STI diagnoses by country

The mean proportions of respondents reporting a diagnosis of syphilis, symptomatic syphilis, gonorrhea, symptomatic gonorrhea, chlamydia, and symptomatic chlamydia in the previous 12 months across all 68 countries, the country median proportion, the country ranges and the IQR are shown in Table 1. The country median was calculated from the 68 country means. The values are adjusted for the French translation artefact by using only the non-French questionnaires in the three European French-speaking countries. Additional Table S1 provides the total numbers of participants from each country, the numbers of self-reported STI diagnoses, and the distribution by symptom status.

**Table 1:** Mean diagnosis rates, country median, country ranges and IQR for self-reported diagnosis of syphilis, gonorrhoea, and chlamydia (overall and symptomatic)

	Mean diagnosis rate (%)	Country median (%)	Range (%)	IQR
Syphilis	4.85	3.92	1.21–10.72	3.22–6.33
Symptomatic syphilis	1.35	1.11	0.0–3.25	0.75–1.41
Gonorrhea	4.46	3.80	1.04–10.02	3.33–5.94
Symptomatic gonorrhea	1.25	1.09	0.0–2.87	0.84–1.49
Chlamydia	3.10	2.15	0.58–11.82	1.55–3.85
Symptomatic chlamydia	0.72	0.54	0.0–2.20	0.34–0.96

Table 2 shows the numbers of self-reported diagnoses, the proportions of how they were classified (symptomatic or asymptomatic), and the reasons for being unclassifiable. There were 415 gonorrhea/chlamydia co-infections classified as symptomatic, which were counted for both, symptomatic gonorrhea and symptomatic chlamydia.

**Table 2:** Classification of self-reported diagnoses and reasons for not being unclassifiable

	Symptomatic	Asymptomatic	Unclassifiable (no recency match)	Unclassifiable (symptomatic co-infection)	Total
Syphilis	27.2% (2442)	18.3% (1637)	51.0% (4577)	3.5% (312)	8968
Gonorrhea	27.3% (2252)	13.2% (1087)	56.6% (4663)	2.9% (238)	8240
Chlamydia	22.6% (1280)	16.8% (950)	58.2% (3298)	2.4% (135)	5663

For a graphical presentation of the respective prevalence and countries covered by this analysis see the UNAIDS key population website [https://kpatlas.unaids.org/dashboard → Men who have sex with men → EMIS/LAMIS data].

The overall ratio of classified symptomatic to total self-reported diagnoses was 1:4 (0.26) for syphilis, slightly higher (0.27) for gonorrhea, and close to 1:5 (0.21) for chlamydia. The correlations between the proportions of respondents by country with classified symptomatic diagnoses and total self-reported diagnoses were high and ranged between r²=0.69 for syphilis, r²=0.68 for gonorrhea, and r²=0.62 for chlamydia (after adjustment for the French translation artefact r²=0.81 for syphilis, r²=0.69 for gonorrhea, and r²=0.65 for chlamydia). Only small numbers of diagnoses and few symptomatic diagnoses were reported from 13 countries with fewer than 450 respondents. Namely, this affects Belarus, Bosnia & Herzegovina, Cyprus, Estonia, Iceland, Latvia, Lebanon, Lithuania, Luxembourg, Malta, North Macedonia, Suriname, and the combined construct of Albania, Kosovo and Montenegro.

The ratio of classified symptomatic to total self-reported diagnoses is influenced by testing patterns, including the proportion of respondents screened for the respective infection, frequency of screenings, type of extragenital sample tested, tests used for diagnosis, and a reporting artefact introduced by a translation issue in the French language questionnaire that affected responses in Europe.

**Fig. 1:**
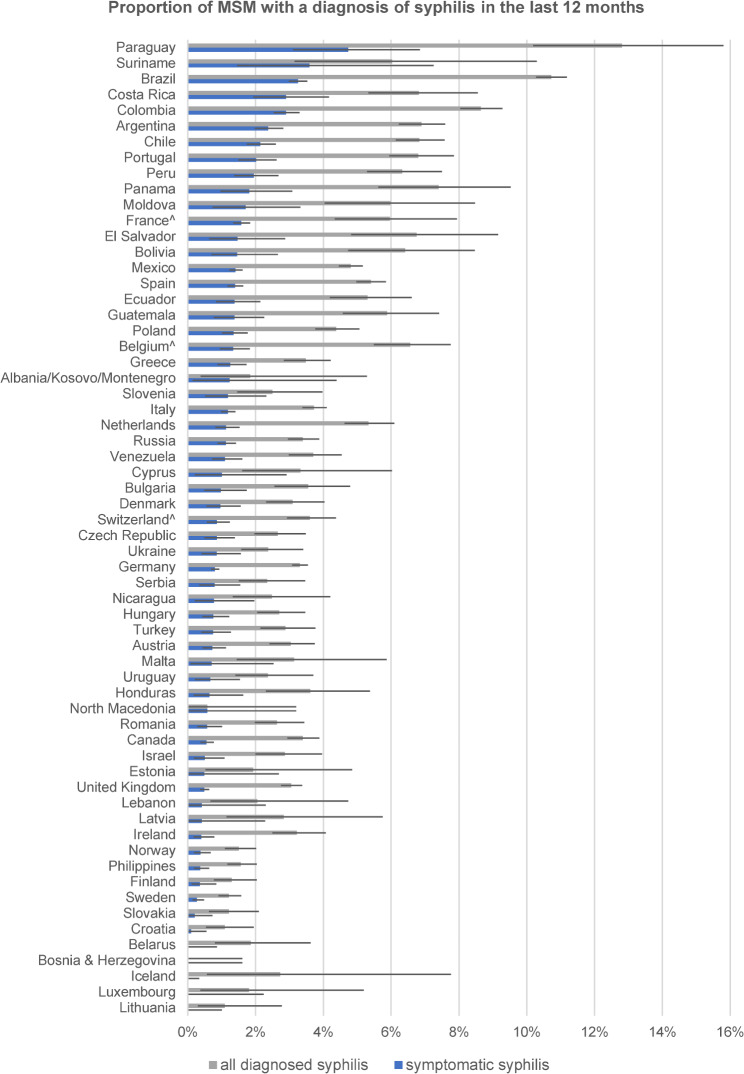
Proportion of MSM by country with overall and symptomatic self-reported syphilis diagnosis in the previous 12 months (including 95% error bars), ranked by proportion with symptomatic syphilis diagnosis

**Fig. 2:**
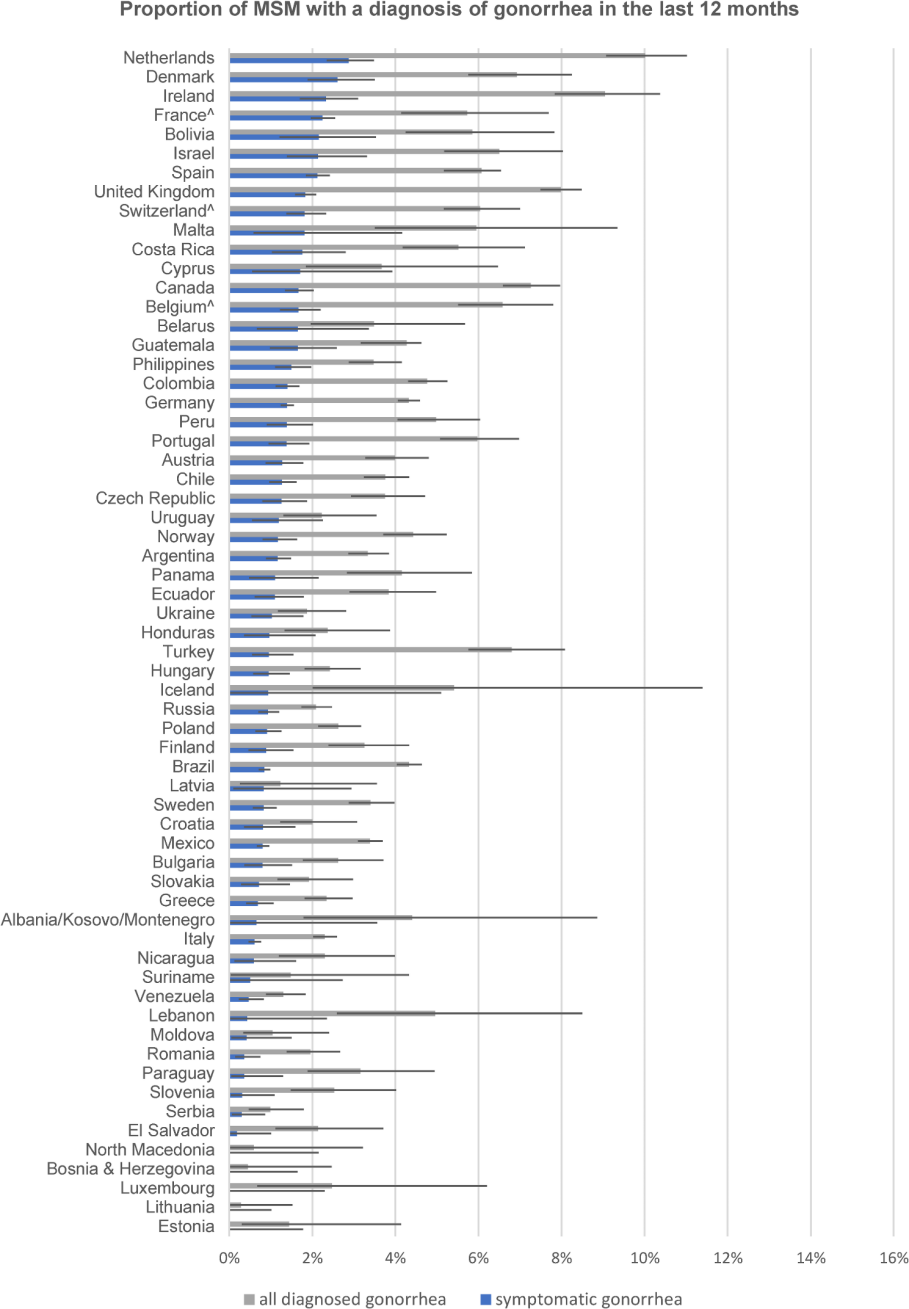
Proportion of MSM by country with overall and symptomatic self-reported diagnosis of gonorrhea in the previous 12 months (including 95% error bars), ranked by proportion with diagnosis of symptomatic gonorrhea

**Fig. 3:**
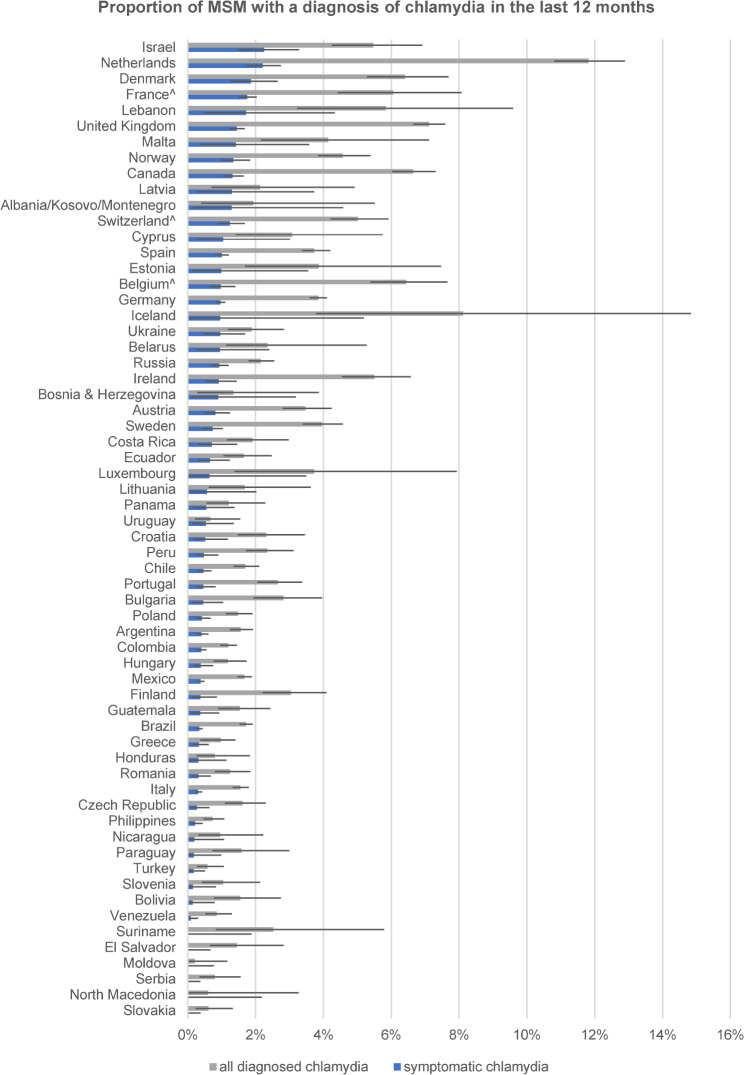
Proportion of MSM by country with overall and symptomatic self-reported diagnosis of chlamydia in the previous 12 months (including 95% error bars), ranked by proportion with diagnosis of symptomatic chlamydia

We used the rate of symptomatic diagnoses to establish a country ranking for the three self-reported STI diagnoses to reduce these potentially large biases (see Figs. 1, 2 and 3). Notably, the rankings were very different for symptomatic syphilis, symptomatic gonorrhea, and symptomatic chlamydia. The rankings for the 13 countries with fewer than 450 respondents should be taken with caution. Potential biases introduced to the ranking by excluding the large numbers of unclassifiable diagnoses will be addressed in the discussion.

Among the top 20 countries for symptomatic syphilis were 14 of 18 countries from Latin America, and six countries (Portugal, Spain, France, Belgium, Poland, and Moldova) from Europe. Among the top 20 countries for symptomatic gonorrhea were only four countries from Latin America (Bolivia, Costa Rica, Guatemala, and Peru), 12 countries from Europe, mostly from western parts, and Israel, Canada, and the Philippines. Among the top 20 countries for symptomatic chlamydia were 17 countries from different parts of Europe, Israel, Lebanon, and Canada, but none from Latin America.

### Behaviors associated with higher risks for STIs

The proportion of respondents reporting more than 10 sex partners in the previous 12 months ranged between 7.9% in Suriname and 45.1% in Israel, the mean across all respondents was 26.5%, the median for all countries was 22.6%. Inconsistent or no condom use with the last non-steady anal intercourse partner was reported by 21.8% of respondents from Sweden and 47.6% from Brazil, the mean across all respondents was 37.1%, and the median for all countries was 36.5%. The lowest proportion reporting multi-partner sex with the last non-steady partner(s) was from Iceland with 3.6% and the highest from the Netherlands with 18.2%. The overall mean for multi-partner sex was 11.0%, the median across countries was 9.9%. Reported HIV PrEP use ranged from 0% in Latvia and Moldova up to 7.8% in the UK, with an overall mean of 2.4% and a country median of 1.7%.

With respect to behaviors associated with higher probabilities of acquiring an STI, two countries from Latin America (Brazil and Argentina), 16 countries from western and southern parts of Europe, and Canada and Israel were among the top 20 countries with the highest proportions of respondents reporting more than 10 sexual partners in the previous 12 months. Ten countries from Latin America and ten countries from western, southern, and eastern parts of Europe reported the largest proportion of inconsistent or no condom use with the last non-steady anal intercourse partner; four countries from Latin America (Colombia, Paraguay, Argentina, and Brazil), the Philippines, Israel and 14 countries from Europe reported the highest proportions of multi-partner sex during the last sex with non-steady partners; two Latin American countries (Peru and Brazil), 15 countries from western parts of Europe as well as Canada, Israel, and Lebanon had the highest proportion taking PrEP.

### Diagnostic procedures and screening

The range for STI testing in the previous 12 months was broad, with the lowest proportion of 18.6% in the Philippines and the highest with 62.2% in the Netherlands. The mean was 46.2%, and the country median 43.1%. The proportion screened within the previous 6 months – used here as a surrogate for screening frequency – ranged from 6.4% in the combined Albania/Kosovo/Montenegro sample to 39.0% in the Netherlands. The mean was 25.2%, and the country median was 23.4%. An anal swab in the previous 12 months was reported by 1.6% of the respondents from Moldova and from 47.6% of respondents from the Netherlands. The mean proportion was 12.4%, and the country median was 10.6%.

Among the top 20 countries regarding the proportion of respondents having been tested for STI in the previous 12 months were 4 countries from Latin America (Brazil, Paraguay, Guatemala, and Chile), Israel, Canada, and 14 countries from all parts of Europe. Among the top 20 countries for having been screened for STI within the previous six months were 4 countries from Latin America (Brazil, Paraguay, Guatemala, and Uruguay), again Israel and Canada, and 14 European countries. Regarding the proportion of respondents having received an anal swab during the previous 12 months, among the top 20 countries there was one country from Latin America (Guatemala), Canada, and 18 countries from Europe, mostly from western parts.

### Multivariable regression models

The multivariable regression models on total and symptomatic self-reported diagnoses yielded similar results for gonorrhea and chlamydia (Tables 3, 4 and 5). This is true for the impact of the French translation issue, major data discrepancies, the impact of HIV diagnosis, screening recency, anal swabbing, partner numbers, and multi-partner sex. The association of screening recency and the probability of a diagnosis shows a clear dose-response relationship with higher diagnosis probabilities at higher testing frequencies. The finding that the medium category is statistically not significant might be surprising but is because the ‘no screening’ reference group comprises individuals who were tested due to symptoms.

**Table 3:** Multilevel multivariable regression model for factors associated with self-reported diagnosis of syphilis (overall and symptomatic) in 68 countries

		Syphilis, all diagnoses						Syphilis, classified symptomatic					
		Univariate analyses, (N = 198,604)			Multivariable analysis (N = 185,729)			Univariate analyses (N = 193,059)			Multivariable analysis (N = 187,364)		
		OR	95% CI	p	aOR	95% CI	p	OR	95% CI	p	aOR	95% CI	p
Region	Central-West Europe (AT, CH, DE, LI, LU)	ref.											
	West Europe (BE, FR, IE, NL, MC, UK)	**1.75**	**1.05–2.93**	**0.033**	0.84	0.48–1.49	0.560	1.22	0.71–2.08	0.467	0.99	0.64–1.52	0.955
	Southwest Europe (AD, ES, IT, MT, PT, SM)	1.46	0.84–2.54	0.177	1.29	0.88–1.91	0.191	**1.86**	**1.06–3.29**	**0.032**	**1.67**	**1.07–2.62**	**0.024**
	Northwest Europe (DK, FI, IS, NO, SE)	**0.51**	**0.29–0.88**	**0.016**	**0.51**	**0.35–0.76**	**0.001**	0.57	0.31–1.05	0.073	0.64	0.38–1.06	0.085
	Northeast Europe (EE, LT, LV)	0.54	0.25–1.16	0.113	0.75	0.40–1.43	0.381	0.32	0.07–1.42	0.133	0.38	0.09–1.64	0.194
	Central-East Europe (CZ, HU, PL, SK, SL)	0.78	0.46–1.34	0.371	1.01	0.70–1.46	0.970	1.14	0.65–2.02	0.647	1.26	0.79–2.02	0.328
	Southeast Europe (AL, BA, BU, CY, HR, GR, HR, ME, MK, RO, RS, TR, XK)	0.69	0.42–1.12	0.132	0.88	0.62–1.24	0.460	0.96	0.56–1.64	0.879	1.06	0.68–1.66	0.785
	East Europe (BY, MD, RU, UA)	0.97	0.55–1.71	0.908	0.87	0.57–1.33	0.526	1.26	0.67–2.35	0.470	1.26	0.75–2.10	0.387
	Middle East (IL, LB)	0.76	0.36–1.62	0.476	0.66	0.37–1.17	0.155	0.62	0.22–1.71	0.356	0.57	0.23–1.41	0.222
	Philippines	0.46	0.20–1.10	0.080	0.57	0.31–1.05	0.072	0.47	0.18–1.25	0.130	0.57	0.25–1.29	0.178
	Canada	1.05	0.46–2.42	0.902	0.56	0.26–1.20	0.137	0.72	0.31–1.71	0.460	0.68	0.34–1.35	0.268
	Mexico	1.48	0.65–3.38	0.346	1.47	0.87–2.51	0.153	1.89	0.85–4.21	0.117	1.63	0.89–2.98	0.117
	Central America (CR, GT, HN, NI, PA, SV)	1.64	0.98–2.74	0.057	**1.86**	**1.29–2.67**	**0.001**	**1.97**	**1.12–3.44**	**0.018**	**2.30**	**1.45–3.66**	**< 0.001**
	Andean region (BO, EC, CO, PE, VE, SR)	**1.84**	**1.11–3.05**	**0.019**	**1.86**	**1.29–2.68**	**0.001**	**2.48**	**1.46–4.22**	**0.001**	**2.42**	**1.57–3.72**	**< 0.001**
	Southern Cone (AR, CL, UY, PY)	**2.04**	**1.18–3.53**	**0.011**	**1.74**	**1.17–2.58**	**0.006**	**3.03**	**1.72–5.33**	**< 0.001**	**2.75**	**1.75–4.32**	**< 0.001**
	Brazil	**3.53**	**1.56–8.02**	**0.003**	2.00	0.94–4.27	0.072	**4.46**	**2.02–9.83**	**< 0.001**	**3.71**	**2.04–6.73**	**< 0.001**
Survey artefacts													
French translation	no	ref.											
	yes	**1.92**	**1.66–2.23**	**< 0.001**	**2.05**	**1.75–2.39**	**< 0.001**	1.09	0.76–1.55	0.645	1.21	0.86–1.71	0.281
Major data discrepancies	no	ref.											
	yes	**1.35**	**1.28–1.43**	**< 0.001**	**1.30**	**1.22–1.38**	**< 0.001**	**1.23**	**1.11–1.37**	**< 0.001**	**1.17**	**1.04–1.31**	**0.008**
Sample composition													
Age group	< 25 years	ref.											
	25–29 years	**1.69**	**1.58–1.80**	**< 0.001**	**1.33**	**1.24–1.43**	**< 0.001**	**1.67**	**1.47–1.89**	**< 0.001**	**1.36**	**1.20–1.55**	**< 0.001**
	30–39 years	**1.95**	**1.84–2.08**	**< 0.001**	**1.30**	**1.21–1.39**	**< 0.001**	**1.92**	**1.71–2.16**	**< 0.001**	**1.38**	**1.22–1.56**	**< 0.001**
	40–49 years	**1.99**	**1.86–2.13**	**< 0.001**	**1.15**	**1.06–1.24**	**0.001**	**1.85**	**1.62–2.12**	**< 0.001**	**1.21**	**1.05–1.40**	**0.008**
	50 + years	**1.64**	**1.52–1.77**	**< 0.001**	0.97	0.89–1.06	0.521	**1.48**	1.26–1.73	< 0.001	**0.99**	0.84–1.18	0.953
Settlement size (inhabitants)	village/countryside (< 10,000)	ref.											
	small town (10,000–99,999)	1.04	0.94–1.14	0.435	1.03	0.93–1.15	0.512	0.96	0.79–1.16	0.653	0.91	0.75–1.11	0.349
	medium town (100,000–499,999)	**1.17**	**1.07–1.28**	**0.001**	1.07	0.97–1.18	0.166	1.09	0.91–1.31	0.354	0.97	0.80–1.16	0.711
	big city (500,000–999,999)	**1.40**	**1.27–1.53**	**< 0.001**	**1.17**	**1.06–1.30**	**0.002**	**1.24**	**1.03–1.49**	**0.023**	1.03	0.85–1.25	0.753
	very big city (≥ 1 million)	**1.59**	**1.46–1.74**	**< 0.001**	**1.16**	**1.05–1.27**	**0.002**	**1.31**	**1.11–1.56**	**0.002**	0.96	0.81–1.15	0.687
Diagnosed HIV	no	ref.											
	yes	**5.27**	**5.05–5.50**	**< 0.001**	**3.84**	**3.65–4.04**	**< 0.001**	**4.11**	**3.78–4.46**	**< 0.001**	**2.96**	**2.70–3.25**	**< 0.001**
Financial coping	comfortable	ref.											
	neither struggling nor comfortable	1.03	0.98–1.07	0.252	1.07	1.02–1.12	0.010	**1.17**	**1.07–1.27**	**0.001**	**1.18**	**1.08–1.30**	**< 0.001**
	struggling	**1.15**	**1.09–1.21**	**< 0.001**	1.10	1.04–1.17	0.001	**1.31**	**1.18–1.45**	**< 0.001**	**1.21**	**1.09–1.36**	**0.001**
Testing behavior													
Last STI screen	no screening	ref.											
	> 6–12 months ago	**0.74**	**0.68–0.80**	**< 0.001**	**0.61**	**0.56–0.67**	**< 0.001**						
	1–6 months ago	**1.54**	**1.46–1.62**	**< 0.001**	0.95	0.90–1.01	0.101						
	within the previous 4 weeks	**2.61**	**2.46–2.77**	**< 0.001**	**1.43**	**1.34–1.53**	**< 0.001**						
Sexual behavior													
Number of sex partners, previous 12 months	none or one	ref.											
	2–4	**1.25**	**1.15–1.34**	**< 0.001**	1.06	0.94–1.18	0.358	**1.30**	**1.11–1.52**	**0.001**	0.94	0.76–1.18	0.617
	5–7	**1.92**	**1.78–2.08**	**< 0.001**	**1.46**	**1.29–1.65**	**< 0.001**	**2.51**	**2.16–2.92**	**< 0.001**	**1.59**	**1.26–2.00**	**< 0.001**
	8–10	**2.26**	**2.07–2.47**	**< 0.001**	**1.57**	**1.38–1.79**	**< 0.001**	**2.89**	**2.44–3.42**	**< 0.001**	**1.71**	**1.34–2.19**	**< 0.001**
	11–20	**2.99**	**2.78–3.21**	**< 0.001**	**1.87**	**1.66 --2.12**	**< 0.001**	**3.58**	**3.10–4.14**	**< 0.001**	**1.91**	**1.52–2.41**	**< 0.001**
	> 20	**5.96**	**5.56–6.38**	**< 0.001**	**2.74**	**2.43–3.09**	**< 0.001**	**5.88**	**5.12–6.77**	**< 0.001**	**2.51**	**1.99–3.16**	**< 0.001**
Anal intercourse, condom use with last non-steady partner (NSP)	no anal intercourse with NSP	ref.											
	never condom with NSP	**3.25**	**2.95–3.58**	**< 0.001**	**1.54**	**1.34–1.77**	**< 0.001**	**3.46**	**2.82–4.24**	**< 0.001**	**1.90**	**1.45–2.49**	**< 0.001**
	seldom condom with NSP	**5.82**	**5.35–6.33**	**< 0.001**	**1.95**	**1.71–2.23**	**< 0.001**	**5.72**	**4.81–6.81**	**< 0.001**	**2.31**	**1.78–2.99**	**< 0.001**
	sometimes condom with NSP	**4.72**	**4.36–5.10**	**< 0.001**	**1.79**	**1.58–2.04**	**< 0.001**	**5.48**	**4.68–6.42**	**< 0.001**	**2.30**	**1.80–2.95**	**< 0.001**
	mostly condom with NSP	**2.93**	**2.73–3.14**	**< 0.001**	**1.41**	**1.25–1.59**	**< 0.001**	**3.80**	**3.29–4.39**	**< 0.001**	**1.87**	**1.47–2.37**	**< 0.001**
	always condom with NSP	**1.34**	**1.24–1.44**	**< 0.001**	**0.83**	**0.73–0.94**	**0.003**	**1.62**	**1.39–1.89**	**< 0.001**	1.00	0.78–1.27	0.977
	no answer condom with NSP	**1.18**	**1.06–1.31**	**0.002**	0.96	0.84–1.10	0.572	**1.31**	**1.05–1.63**	**0.015**	1.05	0.81–1.37	0.705
PrEP use	never / don’t know	ref.											
	when needed	**2.09**	**1.78–2.46**	**< 0.001**	**1.50**	**1.26–1.78**	**< 0.001**	**2.12**	**1.50–2.98**	**< 0.001**	**1.74**	**1.23–2.46**	**0.002**
	former daily use	**2.68**	**2.10–3.42**	**< 0.001**	**2.18**	**1.68–2.82**	**< 0.001**	1.34	0.71–2.52	0.361	1.00	0.51–1.95	0.995
	current daily use	**3.35**	**3.02–3.72**	**< 0.001**	**1.85**	**1.65–2.07**	**< 0.001**	**1.92**	**1.47–2.49**	**< 0.001**	1.14	0.86–1.50	0.364
Multi-partner sex during last NSP sex	no	ref.											
	yes	**2.47**	**2.36–2.60**	**< 0.001**	**1.31**	**1.23–1.38**	**< 0.001**	**2.42**	**2.20–2.66**	**< 0.001**	**1.35**	**1.22–1.49**	**< 0.001**
Country level rates													
Country-level screening rates (Quartiles)	11.28%–	ref.											
	26.57%–	**1.61**	**1.01–2.56**	**0.044**	1.18	0.91–1.52	0.203						
	30.27%–	**1.75**	**1.24–2.46**	**0.001**	1.27	1.00–1.61	0.051						
	41.74–47.34%	**2.56**	**1.57–4.19**	**< 0.001**	1.70	0.95–3.02	0.073						
cons.					**0.01**	**0.01–0.01**	**< 0.001**				**0.00**	**0.00–0.00**	**< 0.001**
Random part													
68 countries^1^	random intercept				**0.04**	**0.02–0.09**					**0.06**	**0.03–0.13**	

^1^ This study includes 68 countries, with four European microstates included in neighboring (Andorra, Liechtenstein) or surrounding (Monaco, San Marino) countries, and with Albania, Montenegro and Kosovo merged to form a region; this results in 62 country-like entities included in the random part of the model

Abbreviations: OR odds ratio; aOR adjusted odds ratio; CI confidence interval; STI sexually transmitted infection; NSP non-steady partner; PrEP pre-exposure prophylaxis

Country labels: AD Andorra; AL Albania; AR Argentina; AT Austria; BA Bosnia-Herzegovina; BE Belgium; BO Bolivia; BU Bulgaria; BY Belarus; CH Switzerland; CL Chile; CO Colombia; CR Costa Rica; CY Cyprus; CZ Czech Republic; DE Germany; DK Denmark; EC Ecuador; EE Estonia; ES Spain; FI Finland; FR France; GR Greece; GT Guatemala; HN Honduras; HR Croatia; HU Hungary; IE Ireland; IL Israel; IS Iceland; IT Italy; LB Lebanon; LI Liechtenstein; LT Lithuania; LU Luxemburg; LV Latvia; MC Monaco; MD Moldova; ME Montenegro; MK Northern Macedonia; MT Malta; NI Nicaragua; NL Netherlands; NO Norway; PA Panama; PE Peru; PL Poland; PT Portugal; PY Paraguay; RO Romania; RS Serbia; RU Russia; SE Sweden; SK Slovakia; SL Slovenia; SM San Marino; SR Surinam; SV El Salvador; TR Turkey; UA Ukraine; UK United Kingdom; UY Uruguay; VE Venezuela; XK Kosovo

**Table 4:** Univariate and multilevel multivariable regression model for factors associated with self-reported diagnosis of gonorrhea (overall and symptomatic) in 68 countries^1^

		Gonorrhea all						Classified symptomatic gonorrhea					
		Univariate analyses (N = 197,843)			Multivariable analysis (N = 182,449)			Univariate analyses (N = 192,315)			Multivariable analysis (N = 186,599)		
		OR	95% CI	p	aOR	95% CI	p	OR	95% CI	p	aOR	95% CI	p
Region	Central-West Europe (AT, CH, DE, LI, LU)	ref.											
	West Europe (BE, FR, IE, NL, MC, UK)	**2.08**	**1.31–3.30**	**0.002**	1.06	0.86–1.31	0.586	**1.56**	**1.02–2.38**	**0.038**	1.26	0.88–1.80	0.207
	Southwest Europe (AD, ES, IT, MT, PT, SM)	1.02	0.62–1.68	0.925	1.20	0.89–1.62	0.241	0.91	0.57–1.45	0.693	0.72	0.48–1.07	0.103
	Northwest Europe (DK, FI, IS, NO, SE)	0.97	0.60–1.58	0.913	0.77	0.58–1.02	0.064	0.89	0.56–1.41	0.616	0.89	0.60–1.32	0.561
	Northeast Europe (EE, LT, LV)	**0.18**	**0.07–0.45**	**< 0.001**	0.55	0.13–2.34	0.414	**0.17**	**0.04–0.74**	**0.018**	**0.17**	**0.04–0.71**	**0.015**
	Central-East Europe (CZ, HU, PL, SK, SL)	**0.57**	**0.35–0.92**	**0.021**	0.95	0.69–1.32	0.780	**0.62**	**0.39–1.00**	**0.050**	**0.55**	**0.36–0.83**	**0.004**
	Southeast Europe (AL, BA, BU, CY, HR, GR, HR, ME, MK, RO, RS, TR, XK)	**0.53**	**0.34–0.82**	**0.004**	0.95	0.68–1.33	0.768	**0.46**	**0.29–0.72**	**0.001**	**0.34**	**0.22–0.51**	**< 0.001**
	East Europe (BY, MD, RU, UA)	**0.44**	**0.26–0.74**	**0.002**	**0.56**	**0.40–0.78**	**0.001**	0.69	0.41–1.16	0.159	**0.46**	**0.29–0.73**	**0.001**
	Middle East (IL, LB)	1.31	0.69–2.48	0.416	**2.43**	**1.58–3.75**	**< 0.001**	1.17	0.59–2.31	0.649	0.78	0.42–1.43	0.417
	Philippines	0.76	0.36–1.62	0.479	0.78	0.48–1.24	0.291	1.07	0.54–2.14	0.841	0.87	0.48–1.56	0.629
	Canada	1.66	0.79–3.48	0.181	0.96	0.69–1.33	0.793	1.20	0.62–2.32	0.592	0.89	0.51–1.54	0.666
	Mexico	0.74	0.35–1.56	0.431	**0.64**	**0.41–0.99**	**0.045**	0.58	0.30–1.11	0.099	**0.39**	**0.22–0.67**	**0.001**
	Central America (CR, GT, HN, NI, PA, SV)	0.74	0.46–1.19	0.219	0.74	0.52–1.05	0.095	0.78	0.48–1.27	0.322	**0.62**	**0.40–0.96**	**0.033**
	Andean region (BO, EC, CO, PE, VE, SR)	0.78	0.49–1.24	0.290	0.81	0.56–1.18	0.276	0.83	0.53–1.29	0.400	**0.58**	**0.40–0.86**	**0.007**
	Southern Cone (AR, CL, UY, PY)	0.68	0.41–1.13	0.140	**0.64**	**0.44–0.94**	**0.023**	0.77	0.48–1.26	0.299	**0.55**	**0.36–0.84**	**0.005**
	Brazil	0.96	0.46–2.01	0.912	0.69	0.45–1.04	0.074	0.60	0.31–1.15	0.124	**0.34**	**0.20–0.58**	**< 0.001**
Survey artefacts													
French translation	no	ref.											
	yes	**1.62**	**1.41–1.87**	**< 0.001**	**1.59**	**1.38–1.83**	**< 0.001**	1.03	0.78–1.37	0.822	0.89	0.68–1.15	0.368
Major data discrepancies	no	ref.											
	yes	**1.28**	**1.20–1.36**	**< 0.001**	**1.20**	**1.12–1.28**	**< 0.001**	1.06	0.94–1.20	0.320	0.98	0.86–1.11	0.710
Sample composition													
Age group	< 25 years	ref.											
	25–29 years	**1.47**	**1.38–1.57**	**< 0.001**	**1.18**	**1.10–1.27**	**< 0.001**	**1.26**	**1.12–1.42**	**< 0.001**	1.06	0.94–1.19	0.375
	30–39 years	**1.44**	**1.35–1.53**	**< 0.001**	1.01	0.94–1.08	0.777	**1.20**	**1.07–1.34**	**0.001**	0.91	0.81–1.02	0.101
	40–49 years	1.04	0.97–1.11	0.287	**0.67**	**0.62–0.73**	**< 0.001**	**0.83**	**0.73–0.95**	**0.005**	**0.57**	**0.50–0.66**	**< 0.001**
	50 + years	**0.57**	**0.53–0.62**	**< 0.001**	**0.42**	**0.38–0.46**	**< 0.001**	**0.48**	**0.40–0.56**	**< 0.001**	**0.36**	**0.30–0.42**	**< 0.001**
Settlement size (inhabitants)	village/countryside (< 10,000)	ref.											
	small town (10,000–99,999)	**1.24**	**1.12–1.38**	**< 0.001**	**1.16**	**1.04–1.29**	**0.010**	**1.48**	**1.20–1.82**	**< 0.001**	**1.38**	**1.12–1.70**	**0.003**
	medium town (100,000–499,999)	**1.71**	**1.55–1.88**	**< 0.001**	**1.38**	**1.24–1.53**	**< 0.001**	**1.84**	**1.51–2.24**	**< 0.001**	**1.61**	**1.31–1.96**	**< 0.001**
	big city (500,000–999,999)	**2.17**	**1.96–2.40**	**< 0.001**	**1.50**	**1.35–1.67**	**< 0.001**	**2.54**	**2.08–3.10**	**< 0.001**	**2.06**	**1.68–2.52**	**< 0.001**
	very big city (≥ 1 million)	**2.72**	**2.48–2.99**	**< 0.001**	**1.66**	**1.50–1.84**	**< 0.001**	**2.94**	**2.44–3.55**	**< 0.001**	**2.28**	**1.88–2.76**	**< 0.001**
Diagnosed HIV	no	ref.											
	yes	**2.06**	**1.96–2.17**	**< 0.001**	**1.36**	**1.27–1.45**	**< 0.001**	**1.38**	**1.23–1.54**	**< 0.001**	1.03	0.91–1.16	0.672
Financial coping	comfortable	ref.											
	neither struggling nor comfortable	0.99	0.95–1.04	0.702	0.99	0.94–1.04	0.748	0.98	0.89–1.07	0.600	0.97	0.89–1.07	0.574
	struggling	1.00	0.94–1.06	0.886	0.96	0.89–1.02	0.173	0.94	0.84–1.05	0.281	0.91	0.81–1.02	0.116
Testing behavior													
Last STI screen	no screening	ref.											
	> 6–12 months ago	**0.50**	**0.45–0.55**	**< 0.001**	**0.32**	**0.29–0.36**	**< 0.001**						
	1–6 months ago	**1.24**	**1.17–1.30**	**< 0.001**	**0.50**	**0.47–0.53**	**< 0.001**						
	within the previous 4 weeks	**2.28**	**2.15–2.42**	**< 0.001**	**0.68**	**0.63–0.73**	**< 0.001**						
Anal swab in the previous 12 months	no	ref.											
	yes	**7.07**	**6.73–7.42**	**< 0.001**	**5.06**	**4.76–5.37**	**< 0.001**						
Sexual behavior													
Number of sex partners, previous 12 months	none or one	ref.											
	2–4	**1.55**	**1.41–1.70**	**< 0.001**	**1.37**	**1.20–1.58**	**< 0.001**	**1.66**	**1.40–1.98**	**< 0.001**	**1.52**	**1.19–1.95**	**0.001**
	5–7	**2.70**	**2.46–2.96**	**< 0.001**	**2.09**	**1.80–2.42**	**< 0.001**	**3.14**	**2.65–3.72**	**< 0.001**	**2.65**	**2.04–3.44**	**< 0.001**
	8–10	**3.51**	**3.18–3.89**	**< 0.001**	**2.46**	**2.11–2.86**	**< 0.001**	**3.79**	**3.15–4.56**	**< 0.001**	**3.01**	**2.29–3.96**	**< 0.001**
	11–20	**5.27**	**4.85–5.74**	**< 0.001**	**3.38**	**2.93–3.91**	**< 0.001**	**4.86**	**4.15–5.69**	**< 0.001**	**3.70**	**2.85–4.80**	**< 0.001**
	> 20	**10.34**	**9.53–11.21**	**< 0.001**	**5.01**	**4.34–5.79**	**< 0.001**	**8.40**	**7.21–9.78**	**< 0.001**	**5.79**	**4.47–7.51**	**< 0.001**
Anal intercourse, condom use with last nonsteady partner (NSP)	no anal intercourse with NSP	ref.											
	never condom with NSP	**3.80**	**3.41–4.23**	**< 0.001**	**1.44**	**1.23–1.69**	**< 0.001**	**3.40**	**2.78–4.17**	**< 0.001**	**1.54**	**1.16–2.03**	**0.003**
	seldom condom with NSP	**7.67**	**6.98–8.42**	**< 0.001**	**1.78**	**1.52–2.07**	**< 0.001**	**5.59**	**4.67–6.69**	**< 0.001**	**1.77**	**1.35–2.33**	**< 0.001**
	sometimes condom with NSP	**6.71**	**6.13–7.34**	**< 0.001**	**1.66**	**1.43–1.92**	**< 0.001**	**5.48**	**4.63–6.49**	**< 0.001**	**1.73**	**1.32–2.25**	**< 0.001**
	mostly condom with NSP	**4.90**	**4.51–5.32**	**< 0.001**	**1.39**	**1.21–1.60**	**< 0.001**	**4.61**	**3.96–5.36**	**< 0.001**	**1.42**	**1.11–1.83**	**0.006**
	always condom with NSP	**2.26**	**2.07–2.47**	**< 0.001**	0.90	0.78–1.04	0.146	**2.33**	**1.99–2.73**	**< 0.001**	0.90	0.70–1.16	0.400
	no answer condom with NSP	**1.30**	**1.15–1.47**	**< 0.001**	**0.84**	**0.71–0.98**	**0.030**	1.22	0.97–1.54	0.088	**0.72**	**0.54–0.96**	**0.024**
PrEP use	never/ don’t know	ref.											
	when needed	**4.68**	**4.13–5.30**	**< 0.001**	**1.63**	**1.41–1.89**	**< 0.001**	**3.58**	**2.80–4.58**	**< 0.001**	**1.95**	**1.51–2.51**	**< 0.001**
	former daily use	**3.83**	**3.10–4.74**	**< 0.001**	**1.70**	**1.33–2.17**	**< 0.001**	**3.10**	**2.03–4.72**	**< 0.001**	**1.81**	**1.18–2.78**	**0.007**
	current daily use	**5.99**	**5.50–6.51**	**< 0.001**	**1.75**	**1.58–1.94**	**< 0.001**	**2.80**	**2.28–3.43**	**< 0.001**	**1.25**	**1.01–1.55**	**0.040**
Multipartner sex during last NSP sex	no	ref.											
	yes	**2.31**	**2.19–2.43**	**< 0.001**	**1.11**	**1.04–1.18**	**0.001**	**1.94**	**1.75–2.15**	**< 0.001**	1.07	0.96–1.19	0.244
Country level rates													
Country-level screening rates (quartiles)	0.81%–	ref.											
	1.92–	0.85	0.60–1.19	0.340	0.87	0.72–1.04	0.116						
	4.28–	1.07	0.72–1.59	0.728	**1.29**	**1.02–1.62**	**0.031**						
	7.63–35.96%	**2.04**	**1.42–2.92**	**< 0.001**	**1.62**	**1.13–2.33**	**0.009**						
Country-level partner notification rates (quartiles)	25.00%–	ref.											
	61.29%–	0.81	0.55–1.20	0.292	**0.49**	**0.39–0.63**	**< 0.001**						
	73.08%–	0.93	0.62–1.41	0.741	**0.48**	**0.37–0.62**	**< 0.001**						
	83.33–100%	1.04	0.68–1.59	0.864	**0.46**	**0.35–0.61**	**< 0.001**						
	cons.				**0.02**	**0.01–0.02**	**< 0.001**				**0.00**	**0.00–0.01**	**< 0.001**
Random part													
68 countries^1^	random intercept				**0.01**	**0.00–0.03**					**0.05**	**0.02–0.10**	

^1^ This study includes 68 countries, with four European microstates included in neighboring (Andorra, Liechtenstein) or surrounding (Monaco, San Marino) countries, and with Albania, Montenegro and Kosovo merged to form a region; this results in 62 country-like entities included in the random part of the model

Abbreviations: OR odds ratio; aOR adjusted odds ratio; CI confidence interval; STI sexually transmitted infection; NSP non-steady partner; PrEP pre-exposure prophylaxis

Country labels: see Table 2

**Table 5:** Univariate and multilevel multivariable regression model for factors associated with self-reported diagnosis of chlamydia (overall and symptomatic) in 68 countries^1^

		Chlamydia all						Classified symptomatic chlamydia					
		Univariate analyses (N = 196,017)			Multivariable analysis (N = 182,449)			Univariate analyses (N = 197,924)			Multivariable analysis (N = 191,658)		
		OR	95% CI	p	aOR	95% CI	p	OR	95% CI	p	aOR	95% CI	p
Region	Central-West Europe (AT, CH, DE, LI, LU)	ref.											
	West Europe (BE, FR, IE, NL, MC, UK)	**2.07**	**1.39–3.07**	**< 0.001**	1.13	0.80–1.58	0.494	1.48	0.96–2.27	0.075	1.18	0.80–1.74	0.416
	Southwest Europe (AD, ES, IT, MT, PT, SM)	**0.63**	**0.41–0.97**	**0.034**	0.74	0.49–1.12	0.156	**0.60**	**0.37–0.98**	**0.041**	**0.53**	**0.34–0.83**	**0.005**
	Northwest Europe (DK, FI, IS, NO, SE)	1.11	0.73–1.68	0.633	0.92	0.64–1.32	0.650	1.04	0.65–1.67	0.872	1.15	0.75–1.77	0.522
	Northeast Europe (EE, LT, LV)	0.55	0.29–1.04	0.068	1.31	0.67–2.53	0.429	0.89	0.37–2.14	0.800	1.09	0.47–2.56	0.842
	Central-East Europe (CZ, HU, PL, SK, SL)	**0.28**	**0.18–0.44**	**< 0.001**	**0.49**	**0.30–0.79**	**0.004**	**0.29**	**0.17–0.52**	**< 0.001**	**0.29**	**0.17–0.50**	**< 0.001**
	Southeast Europe (AL, BA, BU, CY, HR, GR, HR, ME, MK, RO, RS, TR, XK)	**0.32**	**0.21–0.48**	**< 0.001**	**0.58**	**0.35–0.96**	**0.032**	**0.35**	**0.21–0.58**	**< 0.001**	**0.33**	**0.20–0.54**	**< 0.001**
	East Europe (BY, MD, RU, UA)	**0.40**	**0.25–0.65**	**< 0.001**	0.67	0.42–1.05	0.081	0.81	0.47–1.39	0.441	0.71	0.43–1.18	0.187
	Middle East (IL, LB)	1.34	0.76–2.36	0.304	**1.97**	**1.08–3.59**	**0.028**	**2.14**	**1.11–4.11**	**0.023**	1.72	0.94–3.16	0.078
	Philippines	**0.17**	**0.08–0.35**	**< 0.001**	**0.29**	**0.14–0.61**	**0.001**	**0.21**	**0.08–0.56**	**0.002**	**0.22**	**0.09–0.56**	**0.001**
	Canada	1.63	0.86–3.06	0.132	0.98	0.59–1.62	0.932	1.35	0.70–2.63	0.373	1.09	0.60–1.97	0.778
	Mexico	**0.39**	**0.20–0.73**	**0.003**	0.60	0.32–1.11	0.104	**0.38**	**0.19–0.75**	**0.005**	**0.33**	**0.18–0.60**	**< 0.001**
	Central America (CR, GT, HN, NI, PA, SV)	**0.30**	**0.19–0.48**	**< 0.001**	**0.55**	**0.34–0.91**	**0.018**	**0.39**	**0.21–0.72**	**0.003**	**0.39**	**0.21–0.72**	**0.002**
	Andean region (BO, EC, CO, PE, VE, SR)	**0.34**	**0.22–0.52**	**< 0.001**	0.64	0.39–1.06	0.083	**0.34**	**0.21–0.58**	**< 0.001**	**0.30**	**0.19–0.49**	**< 0.001**
	Southern Cone (AR, CL, UY, PY)	**0.33**	**0.21–0.52**	**< 0.001**	0.73	0.42–1.25	0.251	**0.42**	**0.24–0.72**	**0.002**	**0.36**	**0.21–0.59**	**< 0.001**
	Brazil	**0.40**	**0.21–0.75**	**0.004**	0.79	0.42–1.49	0.461	**0.34**	**0.17–0.67**	**0.002**	**0.25**	**0.14–0.46**	**< 0.001**
Survey artefacts													
French translation	no	ref.											
	yes	**1.58**	**1.37–1.83**	**< 0.001**	**1.57**	**1.34–1.83**	**< 0.001**	**1.47**	**1.05–2.05**	**0.024**	1.17	0.87–1.57	0.303
Major data discrepancies	no	ref.											
	yes	**1.24**	**1.15–1.33**	**< 0.001**	**1.18**	**1.08–1.28**	**< 0.001**	1.03	0.88–1.22	0.689	0.96	0.81–1.13	0.610
Sample composition													
Age group	< 25 years	ref.											
	25–29 years	**1.64**	**1.51–1.79**	**< 0.001**	**1.28**	**1.17–1.41**	**< 0.001**	**1.31**	**1.10–1.57**	**0.003**	1.11	0.93–1.33	0.260
	30–39 years	**1.90**	**1.75–2.05**	**< 0.001**	**1.31**	**1.20–1.43**	**< 0.001**	**1.52**	**1.30–1.78**	**< 0.001**	1.13	0.96–1.33	0.153
	40–49 years	**1.56**	**1.43–1.70**	**< 0.001**	1.01	0.92–1.11	0.788	**1.34**	**1.13–1.59**	**0.001**	0.92	0.76–1.10	0.342
	50 + years	1.09	1.00–1.20	0.062	**0.85**	**0.77–0.95**	**0.002**	0.92	0.76–1.11	0.377	**0.68**	**0.56–0.84**	**< 0.001**
Settlement size (inhabitants)	village/countryside (< 10,000)	ref.											
	small town (10,000–99,999)	1.10	0.98–1.22	0.095	1.03	0.92–1.16	0.589	1.04	0.83–1.31	0.717	1.00	0.80–1.26	0.998
	medium town (100,000–499,999)	**1.47**	**1.33–1.63**	**< 0.001**	**1.21**	**1.08–1.35**	**0.001**	**1.30**	**1.05–1.61**	**0.018**	1.17	0.94–1.45	0.167
	big city (500,000–999,999)	**1.79**	**1.61–1.99**	**< 0.001**	**1.20**	**1.07–1.35**	**0.002**	**1.52**	**1.21–1.90**	**< 0.001**	1.22	0.97–1.53	0.085
	very big city (≥ 1 million)	**2.34**	**2.12–2.58**	**< 0.001**	**1.32**	**1.18–1.47**	**< 0.001**	**1.99**	**1.62–2.44**	**< 0.001**	**1.50**	**1.22–1.84**	**< 0.001**
Diagnosed HIV	no	ref.											
	yes	**2.42**	**2.28–2.57**	**< 0.001**	**1.33**	**1.24–1.43**	**< 0.001**	**1.67**	**1.45–1.91**	**< 0.001**	1.15	0.99–1.34	0.072
Financial coping	comfortable	ref.											
	neither struggling nor comfortable	0.96	0.91–1.01	0.137	1.01	0.95–1.07	0.757	0.94	0.84–1.05	0.286	0.98	0.87–1.10	0.719
	struggling	0.96	0.90–1.03	0.279	0.97	0.90–1.05	0.444	0.91	0.78–1.05	0.207	0.90	0.77–1.05	0.194
Testing behavior													
Last STI screen	no screening	ref.											
	> 6–12 months ago	**0.66**	**0.59–0.73**	**< 0.001**	**0.40**	**0.36–0.45**	**< 0.001**						
	1–6 months ago	**1.66**	**1.56–1.76**	**< 0.001**	**0.61**	**0.57–0.66**	**< 0.001**						
	within the previous 4 weeks	**3.03**	**2.84–3.24**	**< 0.001**	**0.81**	**0.74–0.87**	**< 0.001**						
Anal swab in the previous 12 months	no	ref.											
	yes	**8.47**	**8.01–8.95**	**< 0.001**	**5.65**	**5.28–6.05**	**< 0.001**						
Sexual behavior													
Number of sex partners, previous 12 months	none or one	ref.											
	2–4	**1.57**	**1.40–1.76**	**< 0.001**	**1.18**	**1.00–1.39**	**0.044**	**1.64**	**1.31–2.04**	**< 0.001**	1.36	0.99–1.87	0.057
	5–7	**2.56**	**2.28–2.86**	**< 0.001**	**1.56**	**1.31–1.86**	**< 0.001**	**2.43**	**1.94–3.05**	**< 0.001**	**1.82**	**1.30–2.56**	**0.001**
	8–10	**3.64**	**3.23–4.10**	**< 0.001**	**2.05**	**1.72–2.45**	**< 0.001**	**3.50**	**2.76–4.44**	**< 0.001**	**2.46**	**1.73–3.49**	**< 0.001**
	11–20	**5.33**	**4.82–5.89**	**< 0.001**	**2.55**	**2.16–3.02**	**< 0.001**	**4.46**	**3.64–5.46**	**< 0.001**	**2.81**	**2.02–3.93**	**< 0.001**
	> 20	**10.90**	**9.91–12.00**	**< 0.001**	**3.66**	**3.10–4.33**	**< 0.001**	**8.06**	**6.64–9.78**	**< 0.001**	**4.20**	**3.01–5.86**	**< 0.001**
Anal intercourse, condom use with last non-steady partner (NSP)	no anal intercourse with NSP	ref.											
	never condom with NSP	**4.63**	**4.10–5.23**	**< 0.001**	**1.74**	**1.45–2.09**	**< 0.001**	**3.73**	**2.90–4.79**	**< 0.001**	**1.75**	**1.22–2.49**	**0.002**
	seldom condom with NSP	**9.33**	**8.36–10.41**	**< 0.001**	**2.15**	**1.80–2.57**	**< 0.001**	**5.84**	**4.64–7.34**	**< 0.001**	**1.94**	**1.36–2.77**	**< 0.001**
	sometimes condom with NSP	**7.63**	**6.85–8.49**	**< 0.001**	**1.91**	**1.60–2.28**	**< 0.001**	**5.56**	**4.47–6.91**	**< 0.001**	**1.94**	**1.37–2.74**	**< 0.001**
	mostly condom with NSP	**4.94**	**4.47–5.46**	**< 0.001**	**1.51**	**1.27–1.79**	**< 0.001**	**4.52**	**3.72–5.51**	**< 0.001**	**1.69**	**1.21–2.35**	**0.002**
	always condom with NSP	**2.27**	**2.05–2.53**	**< 0.001**	0.99	0.83–1.17	0.911	**2.06**	**1.67–2.54**	**< 0.001**	0.94	0.67–1.31	0.699
	no answer condom with NSP	**1.49**	**1.30–1.72**	**< 0.001**	1.06	0.88–1.27	0.532	1.27	0.95–1.70	0.109	0.84	0.58–1.22	0.360
PrEP use	never/ don’t know	ref.											
	when needed	**4.53**	**3.97–5.18**	**< 0.001**	**1.37**	**1.18–1.60**	**< 0.001**	**3.45**	**2.56–4.66**	**< 0.001**	**1.88**	**1.38–2.57**	**< 0.001**
	former daily use	**4.45**	**3.53–5.61**	**< 0.001**	**1.76**	**1.35–2.29**	**< 0.001**	1.76	0.87–3.56	0.113	1.05	0.52–2.13	0.896
	current daily use	**6.92**	**6.33–7.56**	**< 0.001**	**1.79**	**1.61–1.99**	**< 0.001**	**3.73**	**3.00–4.64**	**< 0.001**	**1.66**	**1.32–2.09**	**< 0.001**
Multipartner sex during last NSP sex	no	ref.											
	yes	**2.41**	**2.27–2.56**	**< 0.001**	**1.11**	**1.04–1.19**	**0.003**	**2.34**	**2.06–2.65**	**< 0.001**	**1.28**	**1.12–1.46**	**< 0.001**
Country level rates													
Country-level screening rates (quartiles)	0.81%–	ref.											
	1.92%–	1.44	1.00–2.07	0.051	**1.33**	**1.01–1.74**	**0.041**						
	4.28%–	**1.72**	**1.13–2.61**	**0.011**	1.26	0.90–1.76	0.177						
	7.63–35.96%	**4.99**	**3.42–7.28**	**< 0.001**	1.54	0.94–2.51	0.086						
	cons.				**0.01**	**0.00–0.01**	**< 0.001**				**0.00**	**0.00–0.00**	**< 0.001**
Random part													
68 countries^1^	random intercept				**0.04**	**0.02–0.09**					**0.05**	**0.02–0.13**	

^1^ This study includes 68 countries, with four European microstates included in neighboring (Andorra, Liechtenstein) or surrounding (Monaco, San Marino) countries, and with Albania, Montenegro and Kosovo merged to form a region; this results in 62 country-like entities included in the random part of the model

Abbreviations: OR odds ratio; aOR adjusted odds ratio; CI confidence interval; STI sexually transmitted infection; NSP non-steady partner; PrEP pre-exposure prophylaxis

Country labels: see Table 2

Stronger differences of associations for the three STIs analyzed were observed for: age groups (stronger decline in older age groups for probability of diagnosis with gonorrhea compared to chlamydia and syphilis); settlement size (stronger increase with increasing settlement size for probability of diagnosis with gonorrhea); condom use (higher probability of diagnosis with chlamydia with declining condom use compared to gonorrhea); and anal swab in the previous 12 months (higher probability of diagnosis with chlamydia with higher proportion of anal swabbing compared to gonorrhea). The impact of the French translation issue is stronger on syphilis than on gonorrhea and chlamydia; age groups affected by an infection are different for gonorrhea and similar for syphilis and chlamydia; having been diagnosed with HIV had a larger impact on syphilis; settlement size and partner numbers were less important. Financial hardship was weakly associated with a syphilis diagnosis; yet this association was not observed with gonorrhea or chlamydia. HIV PrEP use affected the likelihood for a diagnosis with any of the three STIs equally.

In the models for classified symptomatic diagnoses, French language and data discrepancies were no longer significant (or less significant for syphilis); associations with settlement size lost significance for syphilis and chlamydia, but became stronger for gonorrhea; diagnosis probabilities in older age groups decreased for both gonorrhea and chlamydia, and having already been diagnosed and living with HIV ceased to be significant; the impact of partner numbers became stronger, while the impact of condom use remained unchanged. For symptomatic syphilis compared with all syphilis, the impact of age group, financial hardship, HIV diagnosis, partner numbers, and multi-partner sex remained almost unchanged while lack of condom use became more pronounced.

Regions that were particularly affected differed substantially between syphilis and gonorrhea/chlamydia. Compared with Central-West Europe (reference region), most regions in Latin America were more strongly affected by syphilis, while Northwest Europe was least affected. For classified symptomatic syphilis all aORs increased further, and the aOR for Southwest Europe became significant.

Differences between gonorrhea and chlamydia were minor. For both gonorrhea and chlamydia (total diagnoses), the Middle East region (Lebanon and Israel) was more strongly affected than Central-West Europe. For gonorrhea, East Europe, Mexico, and the Southern Cone countries were less affected, Brazil was borderline; for chlamydia, Central East Europe, Southeast Europe, the Philippines and Central America were significantly less affected. East Europe, the Andean region and Mexico were borderline. For classified symptomatic diagnoses of both gonorrhea and chlamydia, significant aORs for the less affected regions declined further and additional regions became significant, such as all other Eastern European regions and the remaining regions in Latin America.

### The impact of partner notification (PN) by people diagnosed with syphilis or gonorrhea

The proportion of respondents reporting PN following a syphilis or gonorrhea diagnosis was categorized in quartiles and entered in the regression models as a country-level variable. In the multilevel models with countries as random component, PN was significantly negatively associated with the diagnosis probability for gonorrhea only, suggesting that PN may indeed interrupt transmission. The probability of a gonorrhea diagnosis was half as likely for men living in countries in the three upper quartiles for gonorrhea PN. However, there was no incremental effect of further increasing PN rates. In the multilevel syphilis model, PN was not associated with a syphilis diagnosis. However, in a multivariable regression model without countries as random component, syphilis PN was inversely associated with the probability of a syphilis diagnosis, with decreasing ORs of a diagnosis and increasing levels of PN. However, aORs decreased in regions with low PN levels when the country-level variable is included in the model (see Additional Table S2). No significant differences were observed regarding the PN variable between the multilevel and a simple multivariable regression model for gonorrhea.

### Assessment of the relative impact of screening activities on the variance of self-reported STI diagnoses

We compared Efrons R² for simple (non-multilevel) logistic regression models with the outcomes syphilis, gonorrhea, and chlamydia diagnosis in the last 12 months including the variables shown in Tables 3, 4 and 5 with Efrons R² for logistic regression models only including screening related variables (syphilis: last STI screen; country-level screening rates; gonorrhea and chlamydia: last STI screen; anal swab; country-level screening rates). For syphilis, the model with the two screening-related variables explained just 15% of the variance explained by the full model (0.013/0.088). For gonorrhea, the model with three screening-related variables explained 58% (0.071/0.122), and for chlamydia 65% (0.088/0.135) of the variance explained by the full models.

## Discussion

We found approximately ten-fold differences between 68 countries on four continents (Europe, North America, South America, Asia) regarding self-reported rates of overall or symptomatic syphilis, gonorrhea and chlamydia diagnoses in the previous 12 months. These findings were partly explained by differences in the national sample compositions, self-reported numbers of sexual partners, condom use for anal intercourse, HIV PrEP use, and the proportion of men reporting sex with multiple partners. For gonorrhea and chlamydia, the extent, frequency and type of STI screening accounted for more than half of the variance in multivariable models. For syphilis, STI screening variables accounted for just 15% of the variance. Country rankings of self-reported total and symptomatic diagnoses are different, reflecting primarily variability in the extent of STI screening practices between countries. Thus, we believe that country rankings for symptomatic self-reported diagnoses – at least for gonorrhea and chlamydia – better reflect disease burden in countries because symptomatic diagnoses should be less impacted by screening practices, and be more relevant for measuring disease burden.

However, our calculations systematically underestimate the proportion of symptomatic diagnoses because we were only able to classify diagnoses that had been diagnosed during the last STI testing, and we also excluded cases when syphilis and gonorrhea or chlamydia were co-diagnosed. The majority of infections that were excluded from our analysis could not be classified with certainty as symptomatic or asymptomatic because they showed discrepancies in the date of the diagnosis and the date of the last STI test. If we assume that unclassifiable infections may distribute similarly to classifiable infections, the burden of self-reported symptomatic infections may approximately at least double.

We assumed that the classification of an infection as symptomatic or asymptomatic is independent of testing recency and that unclassifiable infections would be equally distributed across countries. If distributed equally across countries, the exclusion of unclassifiable infections would therefore not affect the overall relative country ranking. This assumption might look overly simplistic. Since we selected symptomatic diagnoses based on the most recent STI test, this selection could be biased if there is an association between testing frequency and symptoms. Theoretically, and supported by our data, one should expect a higher probability for asymptomatic diagnoses with more frequent testing. Consequently, our ranking would underestimate the proportion of classified symptomatic infections in countries with higher testing frequencies. The correlation between testing frequency (if we use the proportion of respondents tested within the previous month as surrogate for testing frequency) and self-reported prevalence of symptomatic infection was highest for gonorrhea (r^2^ = 0.34), followed by chlamydia (r^2^ = 0.19), and was lowest for syphilis (r^2^ = 0.04). Since countries with high testing frequency generally ranked higher for classified symptomatic gonorrhea and chlamydia, this bias would rank them even higher and thus raise and not lower their individual ranking.

Differences between geographical regions become smaller after controlling for established confounders such as age, having been diagnosed with HIV, partner numbers, condom use, and screening practices. However, some significant differences between regions remain: self-reported syphilis diagnoses were more prevalent in Latin America compared with Europe, Canada, Israel, Lebanon and the Philippines; self-reported gonorrhea diagnoses were less prevalent in East Europe and Latin America compared with the other regions; and self-reported chlamydia diagnoses were less prevalent in Central East and Southeast Europe, Central America, and in the Philippines. For symptomatic diagnoses, these differences in prevalence extend to all Eastern European and Latin American regions.

The higher incidence of syphilis among MSM in Latin America compared with the other regions has also been reported in a recent global systematic review and meta-analysis of studies published from 2000–2020 [40]. This systematic review showed a pooled corrected syphilis prevalence among MSM in Europe and North America of 3.4% (1.8–5.4%) based on studies from 15 countries involving 13,618 MSM with 763 syphilis diagnoses. The finding on Latin America and the Caribbean was based on data from 17 countries and included 32,316 MSM with 4,144 syphilis diagnoses and a pooled corrected prevalence estimate of 10.6% (8.5–12.9%). In our analysis, the median self-reported country prevalence for syphilis was 3.0% in Europe and Canada with a mean overall prevalence of 4.3% among the 130,264 respondents who reported 5,651 diagnoses, and a median self-reported country prevalence of 6.3% in Latin America with a mean overall prevalence of 7.4% among 63,540 respondents who reported 4,727 syphilis diagnoses. Thus, a likely explanation for the different self-reported syphilis prevalence among EMIS and LAMIS participants might be a higher background prevalence of syphilis in Latin America, as highlighted in the recent systematic review findings [40]. This higher background prevalence might possibly be associated with and/or facilitated and enhanced by lower PN rates compared with most European countries.

A recently published systematic review of global gonorrhea prevalence reporting reviewed 2,015 titles and included 174 full-text publications. National population-based prevalence data were identified in only four countries, two of them also included in our samples (UK and Peru). Prevalence data from MSM were identified from 64 studies in 25 countries, but due to the diversity of the studies, detailed comparison across studies was not possible. Rectal infection rates were generally higher than urogenital or pharyngeal infection rates, where extragenital testing was conducted [41].

There are several factors that could explain lower overall self-reported gonorrhea diagnosis rates in Eastern Europe and Latin America in our analysis: lower diagnosis rates could possibly be explained by higher underreporting of STI due to a higher social desirability bias among respondents from these regions, less extragenital testing (which might not have been sufficiently controlled for because we are only able to control for rectal swabbing, not for pharyngeal swabbing), the use of less sensitive tests, and the more common adoption of a syndromic treatment approach [42]. A systematic review on curable STIs in the Americas published in 2015 states that there is “limited availability of reliable and inexpensive STI tests” and “of 18 reporting countries, 16 pursue syndromic management as their national policy” which supports our explanation that syndromic treatment and the use of less sensitive tests may indeed play a role. [43].

However, extragenital testing and the use of less sensitive tests should play a minor role in the differences observed for symptomatic gonorrhea because most pharyngeal and rectal infections with gonorrhea are asymptomatic or present with hardly noticeable symptoms [44–46]; less sensitive tests are usually reactive in symptomatic infections. The effects of syndromic approaches to diagnosis and treatment are less predictable. If syndromic approaches are commonly used to treat symptomatic gonorrhea and chlamydia, it remains uncertain what the respondents in our surveys report because we do not know what they have been told when given syndromic treatment. Healthcare providers might have said they have gonorrhea and/or chlamydia, or they may just have been told they need treatment without being given a proper diagnosis.

Lower diagnosis rates of self-reported symptomatic gonorrhea and chlamydia were reported more often from less affluent countries where access to highly sensitive NAAT diagnostic tools is limited, costs of these tests are prohibitive, and thus syndromic approaches to diagnosis and treatment may be more common. We also cannot exclude that in countries where extragenital screening with NAATs is more common, higher screening levels might have an unintended and not anticipated paradoxical effect on the susceptibility for reinfection that could contribute to higher rates of symptomatic infections with gonorrhea and chlamydia [36, 47]. However, these measures have been implemented despite knowledge gaps on the natural course of asymptomatic infections and resulting immunological reactions. Despite widespread implementation of screening for asymptomatic NG/CT infections in MSM and subsequent treatment, there is currently no evidence for individual or public health benefits of these measures [48, 49]. In any case, it would be sensible to push for evidence of individual and/or public health benefit from gonorrhea and chlamydia screening programs among MSM before attempting to implement such screening, especially in resource-constrained settings

Likewise, low levels of self-reported diagnosed chlamydia in Central East and Southeast Europe, Central America and in the Philippines are possibly explained by less chlamydia-specific (extragenital) testing and/or less sensitive chlamydia tests, and more syndromic treatment of symptomatic infections [42, 50].

The same reasons might have contributed to biased proportions of respondents reporting syphilis, gonorrhea, or chlamydia diagnoses compared with each other. From systematic screening studies on the prevalence of these infections in MSM populations [45, 51–53], we know that gonorrhea and chlamydia are detected at approximately the same frequency, while acute syphilis is detected less frequently. In our samples, syphilis and gonorrhea are reported at similar frequencies, and chlamydia is less frequently reported. Less extragenital testing for chlamydia, the use of less sensitive tests, and higher proportions of asymptomatic chlamydia infections likely explain lower self-reported diagnosis rates for chlamydia compared with gonorrhea. In addition, self-reported syphilis diagnoses may be a mix of acute syphilis and syphilis antibody detection, depending on what has been communicated between healthcare provider and patient. This could increase the number of respondents reporting a syphilis diagnosis. However, this should be less likely for symptomatic syphilis, but we still see similar proportions when comparing rates of symptomatic gonorrhea and symptomatic syphilis. Thus, we rather argue that the similar proportions of syphilis and gonorrhea diagnoses are due to a participation bias in such surveys by men at higher risk for syphilis. From analyses of self-reported HIV prevalence from EMIS-2010, we know that a participation bias of men diagnosed with HIV exists [28]. This participation bias also affects self-reported syphilis prevalence in the sample due to the higher prevalence of syphilis among men diagnosed with HIV.

PN is significant in the multilevel model for gonorrhea with no incremental benefit for further increasing PN rates was detected. This could either suggest that the observed association is confounded by other unknown factors or that positive effects of increasing PN rates are offset by, e.g., adverse effects of increasing screening rates in countries with higher PN rates. Since the date of infection is usually unknown when infections are diagnosed by screening, PN may only be partially effective for interrupting transmission, while potential adverse effects such as arrested immunity development by increased detection and treatment of asymptomatic infections may increase with increasing screening rates.

In the syphilis regression model, the strong association of low PN and high diagnosis levels in Latin America may render it difficult to demonstrate an association in the multilevel model. However, since we can investigate the effect of PN only as a country-level variable, the effect we saw in the simple multivariable regression model might also be confounded by other unknown factors which have a similar country distribution.

### Strengths and limitations

A particular strength of our analysis is the use of the same methods to assess self-reported STI diagnoses in all countries, and the ability to compare diagnoses classified as symptomatic by the respondents. An important strength of the surveys is that we rely on voluntary self-identification of MSM, not on being identified or designated as MSM by others. We restrict our comparisons of self-reported diagnoses for the ranking of countries to diagnoses classified as symptomatic to avoid biases due to different levels of screening in various countries. By using this approach, we also minimize bias from tests with different sensitivity levels since the detection of symptomatic infections depends on this less.

However, relying on self-reported diagnoses also constitutes a major limitation of our analysis. We do not have any information on which testing methods have been used to diagnose infections, which definitions e.g. for active syphilis were used, whether a diagnosis of gonorrhea and/or chlamydia was based on NAAT or less sensitive antigen tests, culture, microscopy, or clinical syndromes. If syndromic approaches have been used, we do not know whether and which kind of diagnosis was communicated to our respondents (e.g. if urethral discharge was diagnosed as gonorrhea, as chlamydia, or none of these). For syphilis, we do not know which proportion of the diagnoses were indeed active infections requiring treatment because we have not asked about subsequent STI treatment.

Being self-reported, diagnosis and behavior data can be subject to social desirability bias, recall bias, erroneous attribution of symptoms to an infection diagnosis, and confusion of different STIs. The question regarding symptoms did not relate to the diagnosed STI, but to the last STI test. We cannot determine the site/location of these infections and do not know whether the symptoms reported by the respondents were related to the infections that had been diagnosed. Theoretically it is possible that symptoms were caused by STIs not queried (e.g. M.genitalium) or not included in this analysis (e.g. anal/genital warts). Our questionnaire did not allow to classify infections as symptomatic or asymptomatic if they had been diagnosed in the previous 12 months, but not during the last STI test. The potential effects of excluding these diagnoses on the ranking exercise have been discussed above. A response to STI screening was missing from 3.6% of questionnaires. Non-response was associated with factors that were also associated with a lower probability for being tested and diagnosed with an STI. We cannot determine how much pharyngeal swabbing has contributed to (mostly asymptomatic) diagnoses of gonorrhea and chlamydia because the questionnaire did not query whether pharyngeal swabs were used. The French translation issue blurred the difference between having been tested for STIs and having been diagnosed with an STI. In comparison with non-French questionnaires, this resulted in a strong and statistically significant overestimation of STI diagnoses among survey participants who completed a French questionnaire. However, no statistical difference was detected between French and other languages when the analysis was restricted to diagnoses classified as symptomatic. This means that diagnosis probability was similar between respondents who reported having been diagnosed with a symptomatic STI and people who responded to a question that was partly understood as having been tested for an STI in the presence of symptoms. We think this argues in favor of a high specificity of reported symptoms for gonorrhea and chlamydia.

Since the survey is a convenience sample, participation could have been affected by lack of access to social networks and technology including the use of smartphones and data differently in the various countries.

## Conclusions

Much of the differences in self-reported STI diagnoses rates across countries is likely explained by differences in testing (i.e. the extent of extragenital screenings for gonorrhea and chlamydia, the proportion of respondents screened for STI, frequency of screening). Further differences can be explained by demographic and behavioral factors, such as age, HIV diagnosis, partner numbers, and condom use for anal intercourse. With our data we are unable to assess the efficacy of extragenital screenings for gonorrhea and chlamydia to reduce the prevalence of these infections among MSM, but we found no indication of beneficial effects of increased screening: most countries with high screening levels among MSM ranked high for self-reported prevalence of symptomatic infections. Before recommending extragenital screening for gonorrhea and chlamydia to MSM in resource-limited settings, individual and/or public health benefit of increased screening should be demonstrated through randomized controlled trials. Further research on the effects of PN as an STI intervention is warranted.

## Electronic supplementary material

Below is the link to the electronic supplementary material.

Supplementary Material 1

Supplementary Material 2

Supplementary Material 3

Supplementary Material 4

## Data Availability

The EMIS-2017 and the LAMIS datasets used for this analysis have been obtained from the London School of Hygiene and Tropical Medicine (EMIS-2017) and from CEEISCAT, Barcelona (LAMIS) under data transfer agreements that prohibit to sharing the datasets publicly. Although we cannot make study data publicly accessible at the time of publication, all authors commit to make the data underlying the findings of the study available in compliance with the BMC Public Health Data Availability Policy. Data requests for the LAMIS dataset should be addressed to CEEISCAT/Fundació IGTP, Ctra. De Canyet s/n, 08916 Badalona – Catalonia, Spain: nlorente@coalitionplus.org, and the first author (MarcusU@rki.de). Data requests for the EMIS-2017 dataset should be addressed to the London School of Hygiene and Tropical Medicine Research Operations Office Data Management Lead: alex.hollander@lshtm.ac.uk, the first author (MarcusU@rki.de), and the Principal Investigator of EMIS-2017 (Peter.Weatherburn@lshtm.ac.uk). Individuals requesting data should present their research objective(s) and enclose a list of requested variables. To protect the confidentiality of participants, data sharing is contingent upon appropriate data handling and good scientific practice by the person requesting the data and should furthermore be in accordance with all applicable local requirements. The London School of Hygiene and Tropical Medicine administrative offices are located at Keppel Street, London WC1E 7HT, United Kingdom.
