# Supplementary material for: Comparison of the burden of self-reported bacterial sexually transmitted infections among men having sex with men across 68 countries on four continents
Source: BMC Public Health. 2023 May 30;23:1008. doi: 10.1186/s12889-023-15946-8 (PMC10228115; doi:10.1186/s12889-023-15946-8)
Supplement: Supplementary file 1 — Supplementary Material 1 [file 12889_2023_15946_MOESM1_ESM.pdf]

Additional File 1

Informed consent for EMIS and LAMIS participation

Welcome to the world's largest survey of gay men, bisexual men and other men (including trans men) who are attracted to men.
(EMIS2017)
Please take part if you are…
a man who has sex with men
or a man who is attracted to other men
or a man who thinks he might have sex with men in the future.
You must be old enough to legally have sex in the country where you live.
What is it about?
It asks about relationships, sex life, risks and precautions, and use of health services.
How long will it take?
Most men take between 15 and 25 minutes to complete it. Please make sure you have enough time and battery power, because you cannot come back to complete it if you log off.
However, the survey is voluntary and you can stop and withdraw at any time. None of the data you supplied will be collected if you do this. Please complete this survey ONLY ONCE this year.
Why should I take part?
Because we all want better sex with less harm. By taking part you might find out something new. Although there will be no direct benefit to you from the information you provide, it will help health and social services to better meet the community's needs. It could also mean that services for gay and bisexual men are funded. Our first version of this survey (in 2010) recruited 181,000 men.
Who is running it?
The survey is being run by Sigma Research in the UK and has been approved by the ethics committee of the London School of Hygiene & Tropical Medicine (University of London).
It has been developed with an international group of researchers and health workers in public health institutes, universities and nongovernmental organisations working in HIV, gay health and human rights, from over 40 European countries.
The survey is funded by the European Commission Health Programme 2014 – 2020 to deliver evidence about the sexual health of gay men, bisexual men and other MSM across Europe. It is supported by the European Centre for Disease Prevention and Control, and many national and international gay organisations and websites.
You can get more information about the survey at www.esticom.eu
What about data protection?
We will **not** collect the TC/IP address
of your device or try to install any cookies on it. We will **not** collect any information about you that would allow anybody to identify you. This means you need to complete the survey in one session and you cannot log out and return later. Participation is voluntary and you can withdraw at any time.
Where will the data go?
The anonymous data will be shared with EMIS academic and community collaborators in participating countries as soon as it is ready. Eventually it will be placed in the European Union Open Data Portal for the use of other researchers after a period of embargo.
When can I see the results?
Results will available after mid 2018 in different formats and languages. They can be accessed at the project's website at www.esticom.eu
Please complete this survey ONCE ONLY this year.
Please answer the following two questions if wish to take part.
Have you read and understood the above information?
COMPULSORY QUESTION
No
Yes
Are you old enough to legally have sex with men in the country you live in?
COMPULSORY QUESTION
No
Yes
I don't know

FORMATO DE CONSENTIMIENTO INFORMADO VIRTUAL

LAMIS 2017: Encuesta on-line para hombres que tienen sexo con hombres en América Latina

¡Bienvenido a esta encuesta orientada a hombres gay, bisexuales y otros hombres que se sienten atraídos por los hombres en América Latina!

Por favor, participe del estudio SOLAMENTE si usted es hombre u hombre transgénero, si tiene por lo menos 18 años de edad, y si:

- tiene sexo con hombres, ó
- se siente atraído por otros hombres, ó

- piensa que podría tener relaciones sexuales con otros hombres en el futuro.

¿Qué es LAMIS 2017?

Es una encuesta anónima, on-line, dirigida a hombres que tienen sexo con hombres en América Latina. Busca proporcionar evidencia sobre su salud sexual y acceso a información y servicios, que pueda ser utilizada para mejorar las políticas públicas y las acciones de las organizaciones enfocadas en esta comunidad. La encuesta recolecta información sobre las relaciones, la vida sexual, los riesgos y precauciones, y el uso de los servicios de salud.

La encuesta es voluntaria. Usted puede detenerse y retirarse en cualquier momento. Si usted se retira antes de terminarla, la información que usted había proporcionado se borrará.

¿Cuánto tiempo me tomará? ¿Puedo comenzarla y continuar después?

La mayoría de los participantes se toman entre 15 y 25 minutos para completar la encuesta.

Como la encuesta es anónima, no se colectan datos identificatorios. Por ello, no se puede comenzar la encuesta y terminarla después. Si usted desea participar en este momento, asegúrese de tener suficiente tiempo (y, si participa desde su celular, suficiente energía de la batería). Si desea participar pero no tiene tiempo en este momento, conserve el enlace para completarla cuando tenga tiempo.

Por favor, para evitar datos duplicados, complete LAMIS 2017 UNA SOLA VEZ.

¿Qué beneficios y riesgos existen en caso de que participe?

Beneficios: Todos queremos que nuestras relaciones sexuales sean más seguras y placenteras, y si usted participa, puede adquirir nuevas perspectivas sobre su sexualidad y su salud. Asimismo, la información que usted proporcione contribuirá a lograr una visión más amplia y actualizada sobre la situación de su comunidad que servirá para generar mejoras en servicios sociales y de salud.

Riesgos: No existen riesgos significativos asociados a su participación, fuera de la incomodidad o ansiedad que algunas preguntas podrían generarle.

Recuerde que su participación es voluntaria y que usted puede retirarse en cualquier momento.

¿Quién organiza y dirige el estudio? ¿Cómo puedo contactar a los responsables?

La encuesta está a cargo del Grupo de Trabajo de LAMIS 2017, que incluye a investigadores de América Latina, con el apoyo de colegas europeos. Si tiene preguntas, puede contactarlos por vía electrónica en las siguientes direcciones: En Perú, Dr. Carlos Cáceres, Universidad Peruana Cayetano Heredia (carlos.caceres@upch.pe); en Chile, Dra. Valeria Stuardo, Universidad de Chile (Valeria.stuardo@gmail.com); y en Brasil, Dra. Maria Amélia Veras, Facultad de Ciencias Médicas de la Santa Casa de Sao Paulo (maria.veras@gmail.com).

El Estudio LAMIS 2017 se basa en la experiencia del Estudio EMIS realizado en línea en Europa (primera fase: EMIS 2010; segunda fase: EMIS 2017).

¿Hay alguna forma de identificarme en la información que se me pide?

La encuesta es ANONIMA. No se pregunta por datos personales que permitan identificar a los participantes. Tampoco se recoge la dirección de TC/IP de su computador o dispositivo, ni se intenta instalar cookies en él (justamente por ello, no hay forma de regresar a la encuesta si se deja sin terminar; se tiene que completar la encuesta en una sesión - no se puede cerrar la sesión y volver más tarde, porque el contenido se perderá).

¿Cómo se almacenará la información? ¿Cómo se analizará?

La información se almacenará de forma segura en un servidor especializado, utilizado por la encuesta europea. Luego de terminado el periodo de respuesta, los datos serán analizados por el equipo de investigadores, con miras a generar: (a) un análisis de información a nivel de América Latina; y (b) análisis de información a nivel de país.

¿Cuándo puedo ver los resultados?

Los resultados estarán disponibles en Diciembre de 2018. Se podrá acceder a ellos en las páginas web de las universidades participantes.

<u>Por favor complete la encuesta LAMIS 2017 una sola vez.</u>

<u>**Si desea participar**, por favor conteste las siguientes dos preguntas</u>:

¿Ha leído y entendido la información anterior? (RESPUESTA REQUERIDA)

( ) No ( ) Sí

¿Tiene 18 años o más? (RESPUESTA REQUERIDA)

( ) No ( ) Sí

¡MUCHAS GRACIAS!
