# Supplementary material for: Comparison of the burden of self-reported bacterial sexually transmitted infections among men having sex with men across 68 countries on four continents
Source: BMC Public Health. 2023 May 30;23:1008. doi: 10.1186/s12889-023-15946-8 (PMC10228115; doi:10.1186/s12889-023-15946-8)
Supplement: Supplementary file 2 — Supplementary Material 2 [file 12889_2023_15946_MOESM2_ESM.pdf]

Additional Fig.S1:
Questions and how they were combined for definition of primary outcomes (and for country level screening variable)

Q149 Have you ever had a test for sexually transmitted infections (STIs) other than HIV?
- 1 No
- 2 Yes
- 3 I don't know

Q150 When did you last have a test for STIs other than HIV?
- 1 Within the last 24 hours
- 2 Within the last 7 days
- 3 Within the last 4 weeks
- 4 Within the last 6 months
- 5 Within the last 12 months
- 6 Within the last 5 years
- 7 More than 5 years ago
- 8 Never
- [recency scale]

Q151 Did you have any symptoms on that occasion?
- 1 No
- 2 Yes
- 3 I don't remember

Q154 Have you provided a blood sample as part of any STI test in the last 12 months?
- 1 No
- 2 Yes
- 3 I don't remember

Q155 Have you provided a urine sample as part of any STI test in the last 12 months?
- 1 No
- 2 Yes
- 3 I don't remember

Q156 Was something inserted into the opening of your penis (urethral swab) as part of any STI test in the last 12 months?
- 1 No
- 2 Yes
- 3 I don't remember

Q160 Was something inserted into your anus (anal swab) as part of any STI test in the last 12 months?
- 1 No
- 2 Yes
- 3 I don't remember

Q162 Have you ever been diagnosed with syphilis?
- 1 No
- 2 Yes
- 3 I don't know
- [If =yes]

Q163 When were you last diagnosed with syphilis?
- [recency scale without never]

Q165 Have you ever been diagnosed with gonorrhea?
- 1 No
- 2 Yes
- 3 I don't know
- [If =yes]

Q166 When were you last diagnosed with gonorrhea?
- [recency scale without never]

Q168 Have you ever been diagnosed with Chlamydia or LGV?
- 1 No
- 2 Yes
- 3 I don't know
- [If =yes]

Q169 When were you last diagnosed with Chlamydia or LGV?
- [recency scale without never]

Definition of **symptomatic diagnoses**:
Q149 = 2; Q150 < 6; Q151 = 2 **and**
Syphilis: Q162 = 2; Q163 = Q150 if both < 6
Gonorrhea: Q165 = 2; Q166 = Q150 if both < 6
Chlamydia: Q168 = 2; Q169 = Q150 if both < 6

Definition of **asymptomatic diagnoses**:
Q149 = 2; Q150 < 6; Q151 = 1 **and**
Syphilis: Q162 = 2; Q163 = Q150 if both < 6
Gonorrhea: Q165 = 2; Q166 = Q150 if both < 6
Chlamydia: Q168 = 2; Q169 = Q150 if both < 6

Definition of **unclassifiable diagnoses**:
Q149 = 2; Q150 < 6 **and**
Syphilis: Q162 = 2; Q163 ≠ Q150
Gonorrhea: Q165 = 2; Q166 ≠ Q150
Chlamydia: Q168 = 2; Q169 ≠ Q150
Or
Symptomatic diagnosis with matching recency for both, syphilis + gonorrhea/chlamydia

Definition of **screening (country-level screening rate** variable):
Q149 = 2; Q150 < 6; Q151 = 1 **and**
Syphilis screening: Q154 = 2
Gonorrhea/chlamydia screening: Q155 or Q156 = 2 **and** Q160 = 2

EMIS-2017 & LAMIS Variable Manual
https://www.emis-project.eu/emis-2017/
