# Supplementary material for: Comparison of the burden of self-reported bacterial sexually transmitted infections among men having sex with men across 68 countries on four continents
Source: BMC Public Health. 2023 May 30;23:1008. doi: 10.1186/s12889-023-15946-8 (PMC10228115; doi:10.1186/s12889-023-15946-8)
Supplement: Supplementary file 3 — Supplementary Material 3 [file 12889_2023_15946_MOESM3_ESM.pdf]

Additional Table S1: Number of respondents per country (in alphabetical order), distribution by self-reported diagnosis, and by assigned symptom status, EMIS-2017 and LAMIS 2018 data.

Codes	no **syphilis**	symptomatic	asymptomatic	unclassifiable	unclassifiable2_3	unknown	Total	no **gonorrhea**	symptomatic	asymptomatic	unclassifiable	unclassifiable2_3	unknown	Total	no **chlamydia**	symptomatic	asymptomatic	unclassifiable	unclassifiable2_3	unknown	Total
Albania/Kosovo/Montenegro	160	2	0	1	0	1	163	152	1	1	5	0	5	159	153	2	0	1	0	1	156
Argentina	5,040	124	65	177	7	184	5,413	5,199	61	11	101	6	107	5,378	5,257	21	3	58	1	59	5,340
Austria	2,588	19	16	43	3	46	2,669	2,554	33	15	57	1	58	2,660	2,533	21	17	51	2	53	2,624
Belarus	423	0	2	6	0	6	431	416	7	3	5	0	5	431	416	4	3	3	0	3	426
Belgium*	1,780	26	16	77	6	83	1,905	1,773	32	15	73	5	78	1,898	1,755	19	15	85	2	87	1,876
Bolivia	671	10	6	28	2	30	717	676	15	5	21	1	22	718	702	1	0	9	1	10	713
Bosnia & Herzegovina	227	0	0	0	0	0	227	223	0	0	1	0	1	224	221	2	1	0	0	0	224
Brazil	15,964	549	373	951	44	995	17,881	17,092	145	93	500	35	535	17,865	17,398	59	57	175	12	187	17,701
Bulgaria	1,113	11	4	23	3	26	1,154	1,117	9	1	19	1	20	1,147	1,102	5	3	22	2	24	1,134
Canada	5,771	32	35	127	13	140	5,978	5,536	95	91	239	8	247	5,969	5,533	75	71	239	9	248	5,927
Chile	4,554	101	61	156	16	172	4,888	4,686	60	11	100	12	112	4,869	4,746	22	8	44	8	52	4,828
Colombia	7,364	224	140	305	28	333	8,061	7,659	109	31	217	26	243	8,042	7,883	31	8	54	2	56	7,978
Costa Rica	930	28	7	29	4	33	998	943	17	7	28	3	31	998	973	7	4	6	2	8	992
Croatia	998	1	1	7	2	9	1,009	978	8	1	9	2	11	998	971	5	4	12	2	14	994
Cyprus	291	3	4	3	0	3	301	289	5	0	6	0	6	300	284	3	1	5	0	5	293
Czech Republic	1,837	16	12	20	2	22	1,887	1,798	23	16	30	1	31	1,868	1,827	5	7	16	2	18	1,857
Denmark	1,630	16	10	24	2	26	1,682	1,559	42	13	59	2	61	1,675	1,563	30	18	58	1	59	1,670
Ecuador	1,339	19	20	35	1	36	1,414	1,354	15	7	31	1	32	1,408	1,369	9	1	13	0	13	1,392
El Salvador	525	8	11	18	1	19	563	549	1	2	8	1	9	561	546	0	2	6	0	6	554
Estonia	204	1	0	3	0	3	208	206	0	0	3	0	3	209	199	2	0	6	0	6	207
Finland	1,372	5	3	10	0	10	1,390	1,337	12	8	25	0	25	1,382	1,338	5	13	24	0	24	1,380
France*	678	9	10	22	2	24	721	675	11	5	24	1	25	716	667	3	10	28	2	30	710
Germany	22,015	179	118	414	42	456	22,768	21,685	307	123	520	29	549	22,664	21,560	211	156	471	25	496	22,423
Greece	2,749	35	12	51	1	52	2,848	2,754	19	8	39	0	39	2,820	2,763	9	1	16	1	17	2,790
Guatemala	1,057	15	19	29	3	32	1,123	1,076	18	2	26	2	28	1,124	1,094	4	3	9	1	10	1,111
Honduras	613	4	5	14	0	14	636	619	6	0	9	0	9	634	629	2	1	2	0	2	634
Hungary	2,095	16	9	27	6	33	2,153	2,096	20	4	22	6	28	2,148	2,099	8	2	13	2	15	2,124
Iceland	107	0	0	2	1	3	110	105	1	1	4	0	4	111	102	1	2	5	1	6	111
Ireland	1,985	8	10	46	2	48	2,051	1,860	45	29	110	1	111	2,045	1,926	18	23	70	1	71	2,038
Israel	1,187	6	5	22	2	24	1,222	1,137	25	10	42	2	44	1,216	1,123	26	7	31	1	32	1,188
Italy	10,467	126	63	210	6	216	10,872	10,577	64	29	149	6	155	10,825	10,567	32	24	108	2	110	10,733
Latvia	240	1	1	5	0	5	247	241	2	0	1	0	1	244	229	3	0	2	0	2	234
Lebanon	238	1	1	3	0	3	243	230	1	4	7	0	7	242	226	4	3	7	0	7	240
Lithuania	362	0	2	2	0	2	366	363	0	0	1	0	1	364	351	2	2	2	0	2	357
Luxembourg	163	0	0	3	0	3	166	158	0	1	3	0	3	162	155	1	1	4	0	4	161
Malta	278	2	3	4	0	4	287	269	5	3	9	0	9	286	278	4	1	7	0	7	290
Mexico	13,971	202	167	323	13	336	14,676	14,165	115	34	340	7	347	14,661	14,291	54	21	159	8	167	14,533
Moldova	456	8	6	15	0	15	485	477	2	1	2	0	2	482	476	0	1	0	0	0	477
Netherlands	3,555	41	29	120	10	130	3,755	3,396	102	52	217	7	224	3,774	3,299	76	77	283	6	289	3,741
Nicaragua	511	4	2	6	1	7	524	510	3	0	8	1	9	522	517	1	1	3	0	3	522
North Macedonia	171	1	0	0	0	0	172	170	0	0	1	0	1	171	167	0	0	1	0	1	168
Norway	2,881	11	3	25	5	30	2,925	2,788	33	18	74	4	78	2,917	2,779	38	16	76	3	79	2,912
Panama	688	13	15	25	2	27	743	716	8	6	15	2	17	747	734	4	1	4	0	4	743
Paraguay	504	26	20	25	3	28	578	552	2	2	11	3	14	570	558	1	0	8	0	8	567
Peru	1,836	37	25	59	3	62	1,960	1,852	26	8	60	3	63	1,949	1,877	9	5	30	1	31	1,922
Philippines	3,283	12	12	26	2	28	3,335	3,224	49	7	58	2	60	3,340	3,276	7	5	12	0	12	3,300
Poland	3,777	52	20	97	4	101	3,950	3,825	35	8	57	3	60	3,928	3,868	16	7	33	2	35	3,926
Portugal	2,344	49	32	81	9	90	2,515	2,346	33	23	86	7	93	2,495	2,383	11	11	39	4	43	2,448
Romania	1,920	11	7	34	0	34	1,972	1,911	7	9	22	0	22	1,949	1,906	6	3	15	0	15	1,930
Russia	5,944	68	35	104	2	106	6,153	5,979	56	14	56	1	57	6,106	5,876	55	21	52	1	53	6,005
Serbia	1,001	8	2	14	0	14	1,025	1,012	3	1	6	0	6	1,022	1,001	0	0	8	0	8	1,009
Slovakia	982	2	2	7	1	8	994	972	7	4	7	1	8	991	978	0	1	5	0	5	984
Slovenia	663	8	4	4	1	5	680	656	2	4	10	1	11	673	666	1	3	3	0	3	673
Spain	9,970	142	83	321	23	344	10,539	9,846	215	90	315	16	331	10,482	9,975	101	62	212	11	223	10,361
Suriname	187	7	1	4	0	4	199	201	1	0	2	0	2	204	193	0	1	4	0	4	198
Sweden	4,330	12	7	32	2	34	4,383	4,208	35	31	81	1	82	4,356	4,180	31	38	101	2	103	4,352
Switzerland*	2,596	21	21	49	6	55	2,693	2,521	52	27	79	4	83	2,683	2,501	32	24	72	4	76	2,633
Turkey	1,721	13	7	30	1	31	1,772	1,644	16	20	83	1	84	1,764	1,720	3	1	5	1	6	1,730
Ukraine	1,153	10	6	11	1	12	1,181	1,157	12	2	7	1	8	1,179	1,147	11	2	9	0	9	1,169
United Kingdom	11,392	56	63	216	24	240	11,751	10,792	204	171	540	21	561	11,728	10,850	161	174	486	10	496	11,681
Uruguay	744	5	5	8	0	8	762	746	9	1	7	0	7	763	748	4	0	1	0	1	753
Venezuela	2,315	26	19	44	0	44	2,404	2,358	11	4	16	0	16	2,389	2,355	2	3	15	0	15	2,375
**Total**	**177,910**	**2,442**	**1,637**	**4577**	**312**	**4,889**	**186,878**	**177,965**	**2,252**	**1,087**	**4,663**	**238**	**4,901**	**186,205**	**178,859**	**1,280**	**950**	**3,298**	**135**	**3,433**	**184,522**

* corrected for French questionnaire artefact
