# Supplementary material for: Comparison of the burden of self-reported bacterial sexually transmitted infections among men having sex with men across 68 countries on four continents
Source: BMC Public Health. 2023 May 30;23:1008. doi: 10.1186/s12889-023-15946-8 (PMC10228115; doi:10.1186/s12889-023-15946-8)
Supplement: Supplementary file 4 — Supplementary Material 4 [file 12889_2023_15946_MOESM4_ESM.pdf]

Additional Table S2: Comparison of Syphilis multilevel multivariable regression model with Syphilis multivariable model, impact of partner notification.

		Syphilis, univariate N=198,604				Syphilis multilevel multivariable, all diagnoses N=184,977				Syphilis multivariable, all diagnoses N=184,977			
		OR	95% CI		p	aOR	95% CI		p	aOR	95% CI		p
**Region**	Central-West Europe	ref.											
	West Europe	**1.75**	1.05	2.93	0.033	0.81	0.45	1.46	0.483	0.86	0.65	1.15	0.312
	Southwest Europe	1.46	0.84	2.54	0.177	1.32	0.90	1.94	0.149	**1.24**	1.09	1.42	0.001
	Northwest Europe	**0.51**	0.29	0.88	0.016	**0.59**	0.39	0.89	0.011	**0.63**	0.51	0.78	<0.001
	Northeast Europe	0.54	0.25	1.16	0.113	0.77	0.40	1.49	0.438	**0.56**	0.33	0.96	0.034
	Central-East Europe	0.78	0.46	1.34	0.371	1.15	0.78	1.69	0.471	**1.49**	1.25	1.78	<0.001
	Southeast Europe	0.69	0.42	1.12	0.132	0.89	0.63	1.27	0.526	0.92	0.79	1.07	0.281
	East Europe	0.97	0.55	1.71	0.908	0.94	0.60	1.48	0.781	**0.69**	0.56	0.86	0.001
	Middle East	0.76	0.36	1.62	0.476	0.73	0.41	1.32	0.299	**0.58**	0.40	0.83	0.003
	Philippines	0.46	0.20	1.10	0.080	0.60	0.31	1.17	0.135	**0.36**	0.26	0.51	<0.001
	Canada	1.05	0.46	2.42	0.902	0.59	0.27	1.29	0.187	0.76	0.54	1.05	0.099
	Mexico	1.48	0.65	3.38	0.346	1.54	0.84	2.82	0.161	0.92	0.77	1.11	0.407
	Central America (CR, GT, HN, NI, PA, SV)	1.64	0.98	2.74	0.057	**2.02**	1.32	3.09	0.001	**1.46**	1.19	1.81	<0.001
	Andean region (BO, EC, CO, PE, and VE & SR)	**1.84**	1.11	3.05	0.019	**1.99**	1.28	3.10	0.002	**1.46**	1.20	1.78	<0.001
	Southern Cone (AR, CL, UY, and PY)	**2.04**	1.18	3.53	0.011	**1.92**	1.22	3.01	0.004	**1.39**	1.15	1.68	0.001
	Brazil	**3.53**	1.56	8.02	0.003	1.85	0.84	4.08	0.126	**1.60**	1.15	2.22	0.005
**Survey artefacts**													
French translation	No	ref.											
	Yes	**1.92**	1.66	2.23	<0.001	**2.03**	1.73	2.37	<0.001	**2.33**	2.03	2.66	<0.001
Discrepant data	No	ref.											
	Yes	**1.35**	1.28	1.43	<0.001	**1.30**	1.22	1.38	<0.001	**1.30**	1.22	1.38	<0.001
**Sample composition**													
Age group	<25 y.	ref.											
	25-29	**1.69**	1.58	1.80	<0.001	**1.33**	1.24	1.43	<0.001	**1.32**	1.23	1.42	<0.001
	30-39	**1.95**	1.84	2.08	<0.001	**1.30**	1.21	1.39	<0.001	**1.29**	1.20	1.38	<0.001
	40-49	**1.99**	1.86	2.13	<0.001	**1.15**	1.06	1.24	<0.001	**1.14**	1.06	1.23	0.001
	50+	**1.64**	1.52	1.77	<0.001	0.97	0.89	1.06	0.494	0.96	0.88	1.05	0.409
Settlement size (inhabitants)	Village/countryside (<10,000)	ref.											
	Small town (10,000–99,999)	1.04	0.94	1.14	0.435	1.04	0.94	1.15	0.507	1.02	0.92	1.13	0.663
	Medium town (100,000–499,999)	**1.17**	1.07	1.28	0.001	1.07	0.97	1.18	0.173	1.05	0.96	1.16	0.288
	Big city (500,000–999,999)	**1.40**	1.27	1.53	<0.001	**1.17**	1.06	1.29	0.002	**1.17**	1.05	1.29	0.003
	Very big city (≥1 million)	**1.59**	1.46	1.74	<0.001	**1.15**	1.05	1.27	0.002	**1.13**	1.03	1.24	0.011
Diagnosed HIV	No	ref.											
	Yes	**5.27**	5.05	5.50	<0.001	**3.84**	3.65	4.03	<0.001	**3.69**	3.50	3.88	<0.001
Financial coping	comfortable	ref.											
	neither struggling nor comfortable	1.03	0.98	1.07	0.252	**1.07**	1.02	1.12	0.010	**1.07**	1.02	1.12	0.009
	struggling	**1.15**	1.09	1.21	<0.001	**1.10**	1.04	1.17	0.001	**1.10**	1.04	1.17	0.002
**Testing behaviour**													
STI screen	No screening	ref.											
	6–12 months ago	**0.74**	0.68	0.80	<0.001	**0.61**	0.56	0.67	<0.001	**0.60**	0.55	0.66	<0.001
	1–6 months ago	**1.54**	1.46	1.62	<0.001	0.96	0.90	1.01	0.107	**0.94**	0.89	0.99	0.023
	Within the last 4 weeks	**2.61**	2.46	2.77	<0.001	**1.43**	1.34	1.52	<0.001	**1.40**	1.31	1.49	<0.001
**Sexual behaviour**													
Number of sex partners, previous 12 months	None or one	ref.											
	2–4	**1.25**	1.15	1.34	<0.001	1.05	0.94	1.18	0.395	1.05	0.94	1.18	0.390
	5–7	**1.92**	1.78	2.08	<0.001	**1.46**	1.29	1.65	<0.001	**1.46**	1.29	1.65	<0.001
	8–10	**2.26**	2.07	2.47	<0.001	**1.57**	1.37	1.79	<0.001	**1.58**	1.38	1.80	<0.001
	11–20	**2.99**	2.78	3.21	<0.001	**1.87**	1.66	2.11	<0.001	**1.86**	1.65	2.10	<0.001
	>20	**5.96**	5.56	6.38	<0.001	**2.73**	2.42	3.08	<0.001	**2.70**	2.39	3.05	<0.001
Anal intercourse, condom use with last non–steady partner	No anal intercourse with non–steady partner	ref.											
	never condom with nsp	**3.25**	2.95	3.58	<0.001	**1.54**	1.34	1.77	<0.001	**1.54**	1.34	1.77	<0.001
	seldom condom with nsp	**5.82**	5.35	6.33	<0.001	**1.96**	1.72	2.24	<0.001	**1.96**	1.72	2.24	<0.001
	sometimes condom with nsp	**4.72**	4.36	5.10	<0.001	**1.80**	1.58	2.04	<0.001	**1.80**	1.58	2.04	<0.001
	mostly condom with nsp	**2.93**	2.73	3.14	<0.001	**1.41**	1.25	1.60	<0.001	**1.42**	1.26	1.60	<0.001
	always condom with nsp	**1.34**	1.24	1.44	<0.001	**0.83**	0.74	0.94	0.003	**0.84**	0.74	0.94	0.004
	no answer condom with nsp	**1.18**	1.06	1.31	0.002	0.96	0.84	1.10	0.597	0.97	0.85	1.11	0.687
PrEP use	never/ don't know	ref.											
	when needed	**2.09**	1.78	2.46	<0.001	**1.49**	1.25	1.77	<0.001	**1.43**	1.20	1.70	<0.001
	former daily use	**2.68**	2.10	3.42	<0.001	**2.18**	1.68	2.83	<0.001	**2.09**	1.61	2.70	<0.001
	current daily use	**3.35**	3.02	3.72	<0.001	**1.85**	1.65	2.07	<0.001	**1.76**	1.57	1.97	<0.001
Multipartner sex	no	ref.											
	yes	**2.47**	2.36	2.60	<0.001	**1.31**	1.23	1.38	<0.001	**1.31**	1.24	1.38	<0.001
**Country level rates**													
Country-level screening rates	11.28% -	ref.											
	26.57%-	**1.61**	1.01	2.56	0.044	1.11	0.86	1.43	0.422	1.11	0.99	1.25	0.081
	30.27%-	**1.75**	1.24	2.46	0.001	1.18	0.91	1.53	0.208	**1.16**	1.03	1.31	0.011
	41.74%- 100%	**2.56**	1.57	4.19	<0.001	**1.86**	1.00	3.46	0.050	**1.58**	1.16	2.15	0.004
Partner notification rates after syphilis diagnosis	0.00% -	ref.											
	53.94% -	1.35	0.91	2.00	0.131	1.03	0.81	1.31	0.814	**0.82**	0.74	0.92	0.001
	67.98% -	1.00	0.68	1.49	0.984	1.17	0.84	1.61	0.356	**0.65**	0.56	0.76	<0.001
	78.76% - 100%	**0.66**	0.44	0.99	0.044	0.91	0.62	1.32	0.610	**0.48**	0.40	0.58	<0.001
constant						**0.01**	0.01	0.01	<0.001	**0.01**	0.01	0.02	<0.001
**Random part**													
68 countries[1]	Random Intercept					**0.05**	0.03	0.09					

1 This study includes 68 countries, with four European microstates included in neighbouring (Andorra, Liechtenstein) or surrounding (Monaco, San Marino) countries, and with Albania, Montenegro and Kosovo merged to form a region; this results in 62 country-like entities included in the random part of the model.
